# Novel Inhibitors of Acetyl- and Butyrylcholinesterase Derived from Benzohydrazides: Synthesis, Evaluation and Docking Study

**DOI:** 10.3390/ph16020172

**Published:** 2023-01-24

**Authors:** Neto-Honorius Houngbedji, Šárka Štěpánková, Václav Pflégr, Katarína Svrčková, Markéta Švarcová, Jarmila Vinšová, Martin Krátký

**Affiliations:** 1 Department of Organic and Bioorganic Chemistry, Faculty of Pharmacy in Hradec Králové, Charles University, Akademika Heyrovského 1203, 500 05 Hradec Králové, Czech Republic; 2 Department of Biological and Biochemical Sciences, Faculty of Chemical Technology, University of Pardubice, Studentská 573, 532 10 Pardubice, Czech Republic

**Keywords:** acetylcholinesterase, benzohydrazide, butyrylcholinesterase, hydrazine-1-carboxamide, enzyme inhibition, molecular docking

## Abstract

On the basis of previous reports, novel 2-benzoylhydrazine-1-carboxamides were designed as potential inhibitors of acetylcholinesterase (AChE) and butyrylcholinesterase (BChE). Inhibitors of these enzymes have many clinical applications. 2-(Substituted benzoyl)hydrazine-1-carboxamides decorated with *N*-methyl or tridecyl were prepared with three methods from commercially available or self-prepared hydrazides and isocyanates. For methyl derivatives, *N*-succinimidyl *N*-methylcarbamate was used or methyl isocyanate was prepared via Curtius rearrangement. Tridecyl isocyanate was synthesized again via Curtius rearrangement or from triphosgene and tridecylamine. The compounds were evaluated for the inhibition of AChE and BChE using Ellman’s spectrophotometric method. Most of the derivatives showed the dual inhibition of both enzymes with IC_50_ values of 44–100 µM for AChE and from 22 µM for BChE. In general, the carboxamides inhibited AChE more strongly. A large number of the compounds showed better or quite comparable inhibition of cholinesterases in vitro than that of the drug rivastigmine. Molecular docking was performed to investigate the possible conformation of the compounds and their interactions with target enzymes. In both AChE and BChE, the compounds occupied the enzyme active cavity, and, especially in the case of BChE, the compounds were placed in close proximity to the catalytic triad.

## 1. Introduction

2-Acylhydrazine-1-carboxamides are a group of compounds with interesting and potentially useful biological properties utilized in drug design. They have been reported as antimycobacterial [1,2], antibacterial [3,4], antifungal [4], anticancer [5], anticonvulsant [6] and anti-inflammatory [3] agents. Importantly, hydrazine-1-carboxamides have also been reported as inhibitors of cholinesterases [2,7].

**Figure 1:**
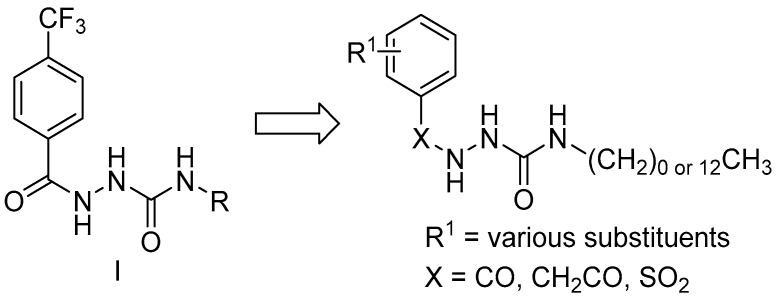
Design of novel cholinesterase inhibitors.

Krátký et al. [2] reported a series of *N*-alkyl-2-[4-(trifluoromethyl)benzoyl]hydrazine-1-carboxamides (I, Figure 1; *n*-alkyl chain R from C_1_ to C_18_) as inhibitors of acetylcholinesterase (AChE) and butyrylcholinesterase (BChE). All hydrazinecarboxamides exhibited dual inhibition of both AChE and BChE, with IC_50_ values of 27.0–106.8 μM and 58.0–277.5 μM, respectively. In the case of AChE inhibition, the IC_50_ values of the most potent inhibitors were lower than those of the established drug rivastigmine. The most potent and also selective AChE inhibitors were found to be *N*-tridecyl/pentadecyl-2-[4-(trifluoromethyl)benzoyl]hydrazine-1-carboxamides. *N*-Methyl analog was identified as the third most potent AChE inhibitor along with significant inhibition of BChE. Molecular docking studies suggest that these compounds may act as non-covalent inhibitors located near the catalytic triad.

Given together with the fact that AChE and/or BChE inhibitors are a cornerstone of therapy for neurodegenerative diseases, including Alzheimer’s disease, myasthenia gravis [8] and other diseases, we designed and conducted a follow-up study of potential cholinesterase inhibitors based on the most potent *N*-methyl/tridecyl-2-[4-(trifluoromethyl)benzoyl]hydrazine-1-carboxamides. The changes performed included the type of substituent on the benzene ring and its position, as well as other types of amide linkers (Figure 1).

## 2. Results and Discussion

### 2.1. Design

Based on the successful hydrazinecarboxamide scaffold (I, Figure 1; compounds **P1** and **P2**), we decided to retain *N*-methyl and *N*-tridecyl chains. *N*-Tridecyl was selected and preferred over *N*-pentadecyl moiety. Both of these alkyls exhibit similar activity, but tridecyl derivatives are less lipophilic and, based on in silico calculations, some pentadecyl derivatives would exceed the log*P* value of 5. However, even among our compounds, there are tridecyl derivatives with higher log*P* values.

Focusing on the benzene ring, first we replaced the 4-CF_3_ group using diverse substituents (series 1) with different electronic (+I, −I, +M, −M), steric (small, bulkier, planar), lipophilic and hydrogen bonding properties. We included ethers (OCF_3_: **1a**, **1b**; OCH_3_: **1u**, **1v**; OPh: **1w**, **1x**), all halogens (**1c**–**1j**), nitro compounds (**1k**, **1l**), nitriles (**1m**, **1n**), alkyls (Me: **1o**, **1p**; *tert*-butyl: **1q**, **1r**), phenyl (**1s**, **1t**), dimethylamino (**1y**, **1z**), hydroxy (**1aa**, **1bb**) and sulfonamido (**1cc**, **1dd**) moieties, as well as unsubstituted benzene (**1ee**, **1ff**) for comparison.

Based on the results of this first series, we designed series 2 covering the positional isomers of the most potent AChE inhibitors (**P1**, **P2** → **2a**–**2d**) and BChE inhibitors (4-NO_2_ group: **1l** → **2e**–**2h**; 4-bromine: **1h** → **2i**–**2l**).

The last issue for modification was the linker connecting the 4-(trifluoromethyl)phenyl scaffold to the hydrazinecarboxamide (series 3). The C=O group was replaced by sulfonyl (**3c**, **3d**), or one methylene group was inserted between CO and the benzene ring (**3a**, **3b**).

### 2.2. Synthesis

The most common method for the preparation of 2-benzoylhydrazine-1-carboxamides and their analogs with modified linkers is the reaction of corresponding arylhydrazides with isocyanates.

**Scheme 1:**
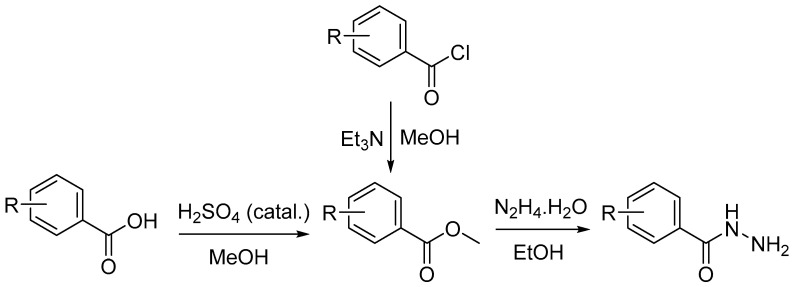
Synthesis of benzohydrazides.

The hydrazides that we used were commercially available or prepared in-house (Scheme 1; 4-iodobenzohydrazide, [1,1’-biphenyl]-4-carbohydrazide, 4-phenoxybenzohydrazide, 4-(trifluoromethoxy)benzohydrazide, 2- and 3-(trifluoromethyl)benzohydrazides, 4-(hydrazinecarbonyl)benzenesulfonamide, 4-cyanobenzohydrazide, as well as hydrazides with modified linkers, 2-phenylacetohydrazide and 4-(trifluoromethyl)benzenesulfonylhydrazide). Their syntheses started from free acids, which were almost quantitatively converted to methyl ester via Fischer esterification (with sulfuric acid as a catalyst). The esters were reacted under reflux with an excess of hydrazine hydrate to give corresponding hydrazides. Alternatively, when available, substituted benzoyl chloride was converted to methyl ester using methanol in the presence of triethylamine as a tertiary base.

**Scheme 2:**
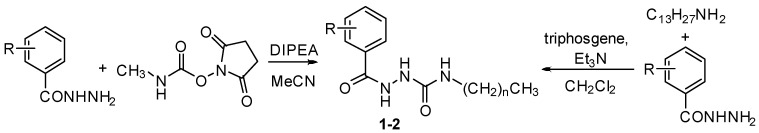
Synthesis of *N*-methyl/tridecyl-2-benzoylhydrazine-1-carboxamides **1–2** (n = 0 or 12, R = H, 4-F, 4-Cl, 2-/3-/4-Br, 4-I, 2-/3-CF_3_, 4-OH, 4-Me, 4-Ph, 4-*t*-Bu, 4-OMe, 2-/3-/4-NO_2_, 4-(CH_3_)_2_N; DIPEA = *N*,*N*-diisopropylethylamine).

Methyl isocyanate and tridecyl isocyanate are not routinely commercially available. Due to the notorious toxicity of methyl isocyanate [9], *N*-methylated hydrazinecarboxamides were initially prepared by reacting hydrazides with an excess of *N*-succinimidyl *N*-methylcarbamate (also known as “methyl isocyanate substitute”) in the presence of *N*-ethyl-*N*,*N*-diisopropylamine (DIPEA) as a non-nucleophilic tertiary base (method A; Scheme 2) [2]. Tridecyl isocyanate, as a compound with an odd number of carbons in the alkyl chain, was firstly prepared in situ from tridecylamine using triphosgene in the presence of triethylamine under a nitrogen atmosphere. Then, it was reacted with hydrazide to afford target *N*-tridecylhydrazinecarboxamides (method B; Scheme 2). *N*-Alkyl-2-{2-[4-(trifluoromethyl)phenyl]acetyl}hydrazine-1-carboxamides **3a** and **3b** were also obtained in this way.

**Scheme 3:**
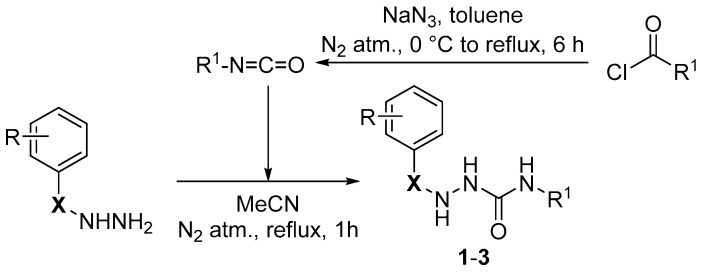
Synthesis of *N*-methyl/tridecylhydrazine-1-carboxamides **1–3** using Curtius rearrangement (R = 3-NO_2_, 4-CN, 4-OCF_3_, 2-/3-/4-CF_3_, 4-OPh, 4-SO_2_NH_2_; R^1^ = CH_3_ or CH_2_(CH_2_)_11_CH_3_; X = CO, SO_2_).

However, both these methods gave inconsistent and generally lower yields (Table 1). Therefore, we decided to prepare both isocyanates via the Curtius rearrangement from commercially available chlorides (acetyl and myristoyl chloride), which were converted to azides via reaction with sodium azide in toluene and were then gently heated to induce rearrangement and thus the formation of isocyanates [10]. The crude isocyanate was immediately used for a subsequent reaction with hydrazides (method C; Scheme 3). This procedure led to significantly increased yields (Table 1); lower yields were in the case of nitro- and sulfamoylbenzohydrazide derivatives.

Compounds **1–3** (Table 2) were characterized by NMR (^1^H, ^13^C) and IR spectra and melting points. The purity was verified additionally by elemental analysis.

### 2.3. Inhibition of Acetylcholinesterase and Butyrylcholinesterase

Hydrazinecarboxamides **1–3** were examined in vitro for their potential to inhibit the function of AChE (obtained from electric eel) and BChE from equine serum using Ellman’s spectrophotometric method (Table 2). The activities are expressed as IC_50_ values, i.e., concentrations leading to 50% inhibition of enzymatic activity. Based on the IC_50_ values for AChE and BChE, we calculated selectivity indexes (SI) that quantified the selectivity for each cholinesterase. SI is the ratio of IC_50_ for BChE/IC_50_ for AChE. Values less than 1 indicate the preferential inhibition of BChE, whereas values above 1 mean stronger inhibition of AChE. For comparison, the clinically used drug rivastigmine, a dual inhibitor of AChE and BChE, was employed.

Most of derivatives **1–3** produced a duality of inhibition of both cholinesterases, although with different potencies. Only **1i** together with some derivatives from series 2, i.e., the positional isomers of the most potent inhibitors from series 1, did not significantly inhibit BChE (IC_50_ >500 μM; 2- and 3-nitro compounds **2e** and **2h**, 2- and 3-brominated analogs **2j**–**2l**). AChE was inhibited with a narrower range of IC_50_ values (44.08–100.08 µM) with the most potent inhibitors being 2-(4-chlorobenzoyl)-*N*-methylhydrazine-1-carboxamide **1e** and 2-(3-bromobenzoyl)-*N*-tridecylhydrazine-1-carboxamide **2j**. In contrast, the lowest activity was associated with 2-(4-methylbenzoyl)-*N*-tridecylhydrazine-1-carboxamide **1p**. The replacement of the 4-CF_3_ group with any substituent (series 1) or its positional isomers (series 2) did not result in improved AChE inhibition. Consistently, better inhibition was associated with electron-withdrawing 4-NO_2_ and 4-CN groups (**1k**–**1n**) and 2-/3-Br (**2i**-**2l**). In series 1, less lipophilic *N*-methyl derivatives are predominantly more potent AChE inhibitors than tridecyl compounds; the exceptions are derivatives with large aromatic 4-phenyl(oxy) moieties (**1s**, **1t** and **1w**, **1x**) and sulfonamides **1cc** and **1dd**. Focusing on positional isomers (series 2), the activity dropped (CF_3_: **P1** and **P2** vs. **2a**-**2d**; NO_2_: **1k** and **1l** vs. **2e**–**2g**). For bromine, the situation was not uniform. For *N*-methyl analog **1g**, both 3- and 2-Br isomers (**2i** and **2k**) did not provide an increase in activity, but among tridecylated hydrazinecarboxamides, this modification resulted in lower IC_50_ values (**1h** vs. **2j** and **2l**). Focusing on linker modifications (series 3), the replacement of C=O with both CH_2_CO and SO_2_ was detrimental (**P1**, **P2** vs. **3a**–**3d**), favoring original benzohydrazides.

In the case of BChE inhibition, the results are quite different. This hydrolase was inhibited in a wider concentration range (IC_50_ of 22.31–>500 µM). Based on the IC_50_ values, hydrazinecarboxamides can be divided into three groups: (1) highly active inhibitors (IC_50_ values of 22.31–88.68 µM) comprising *N*-tridecyl derivatives of 4-nitro- (**1l**), 4-bromo- (**1h**), 4-phenyl- (**1t**), 4-*tert*-butyl- (**1r**) and 4-chlorobenzohydrazide (**1f**); (2) the largest group of compounds with moderate activity (IC_50_ values of 125.23–483.50 µM); and (3) inactive compounds with IC_50_ values above 500 μM (**1i**, **2e**, **2h** and **2j**–**2k**). The positional isomers clearly led to a reduction in or abolition of the activity (series 1 vs. series 2), as did a linker modification (series 1 vs. series 3). Only changes in the 4-substitution of benzohydrazide showed a positive effect on the inhibition of BChE, which mainly favored the nitro group (**1k**, **1l**). For most *N*-methyl vs. tridecyl pairs in series 1, a longer alkyl was beneficial (up to 13.3× for **1g** vs. **1h**).

Concerning the selectivity to both enzymes (SI in Table 2), most of the derivatives were preferential AChE inhibitors (SI values of 1.9–>11.3). The most selective inhibitors of AChE were derivatives of 2- and 3-bromobenzohydrazide (**2j**–**2l**), 2-nitrobenzohydrazide (**2h**) and sulfonamide **3d** with SI > 8.4. Three tridecylated compounds were balanced inhibitors of both enzymes (**1f**, **1r** and **1t**), and only two hydrazinecarboxamides inhibited BChE more strongly than AChE: *N*-tridecyl derivatives of 4-bromo- and 4-nitrobenzohydrazide **1h** and **1l**. Comparing parent molecules **P**, series 1 and 2, the positional isomers of the 4-substituted benzohydrazides were more selective to AChE.

When compounds **1**, **2** and **3** were compared with rivastigmine, some of them exhibited lower IC_50_ values than this drug, namely **1e**, **1g**, **2i** and **2j**, and many of them showed comparable activity (**1a**, **1c**, **1d**, **1k**-**1o**, **1q**, **1t**, **1u**, **1x**, **2a**, **2c**, **2h**, **2k**, **2l** and **3d**). Three *N*-tridecyl derivatives were better inhibitors of BChE than rivastigmine (**1h**, **1l** and **1t**). In this respect, two compounds generally showed better inhibitory properties in vitro than those of rivastigmine: 2-(4-nitrobenzoyl)-*N*-tridecylhydrazine-1-carboxamide **1l** and 2-(4-phenylbenzoyl)-*N*-tridecylhydrazine-1-carboxamide **1t**, which are more potent BChE inhibitors and are also slightly better AChE inhibitors. Keeping in mind the design of the study to improve the activity of *N*-methyl/tridecyl-2-[4-(trifluoromethyl)benzoyl]hydrazine-1-carboxamides **P1** and **P2** [2], the goal for the inhibition of AChE was not achieved, but for BChE, many tridecyl derivatives presented here were superior to the parent compound **P2** (**1b**, **1f**, **1h**, **1j**, **1l**, **1r**, **1t**, **1z**, **1bb**, **1dd**, **1ff** and **2f**), even 12.4× for 4-nitrobenzohydrazide derivative **1l**.

### 2.4. Molecular Docking

The active site of AChE has been investigated thoroughly by AChE mutant studies and X-ray crystallography of the enzyme bound to various ligands [11,12,13].

The hydrolysis of acetylcholine takes place at the bottom of a deep and narrow gorge and is directly mediated by three amino acids, Ser203, His447 and Glu334, forming the so-called catalytic triad. However, several important regions, responsible for the navigation of the substrate to the gorge and its suitable orientation in the cavity, have been described. The so-called peripheral anionic site, formed mostly by aromatic residues (Trp286, Tyr124, Tyr72 and Tyr341), is important for the formation of cation–π interactions with cationic substrates, which then proceed toward a choline-binding pocket (Trp86 and Tyr337) and acyl-binding pocket (Phe295, Phe297, and Trp236), which is believed to be responsible for accommodating the acyl part of the substrate after hydrolysis takes place [14].

A comparison of the active sites of AChE and BChE revealed that most differences in the substrate’s specificity originate from the fact that many aromatic residues present in the AChE active site are missing in BChE, being replaced by smaller hydrophobic residues. Out of 10 aromatic residues interacting with ligands in AChE, only 4 remain in BChE: Tyr332 (Tyr341 in AChE) in the peripheral site and Trp82, Phe329 and Trp231 (Trp86, Phe338 and Trp236 in AChE) in the active site [14]. This, among other things, allows more spacious ligands to enter the cavity.

To better understand the binding mode of the prepared compounds in AChE and BChE, a molecular modeling study was performed.

**Figure 2:**
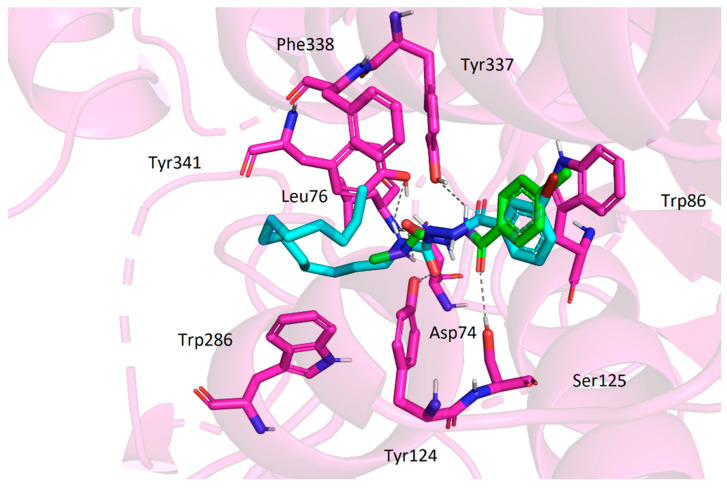
Conformation of **1e** (green) and **2j** (cyan) in the active site of AChE and surrounding amino acid residues.

The top scoring docking pose of two most potent AChE inhibitors in the series, **1e** and **2j**, gave a similar orientation of these compounds in the cavity of the enzyme despite quite large differences in their structures (Figure 2). The substituted aryl moiety showed stacking with Trp86, and the 2-acylhydrazine-1-carboxamide linker was stabilized in its position by several H-bond interactions (namely with Asp74, Tyr124, Ser125 and Tyr31). In the case of compound **2j**, the long tridecyl tail was heading out toward the entrance of the cavity, where several hydrophobic amino acid residues were (Leu76, Phe338 and Trp286), which further contributed to the additional stabilization of the ligand in the enzyme.

**Figure 3:**
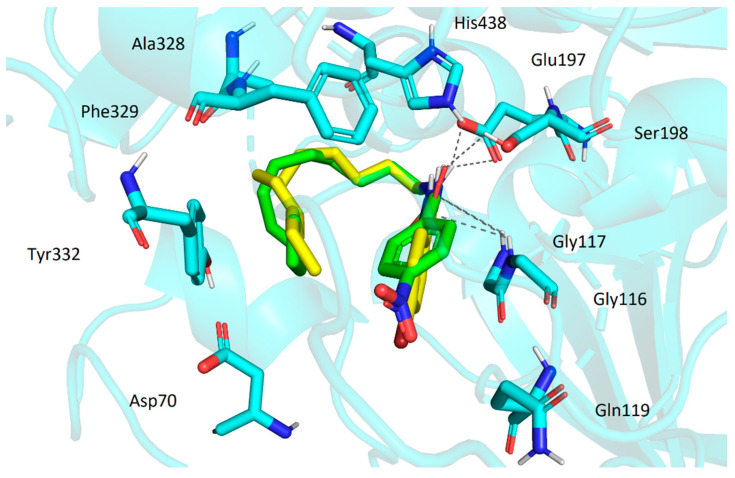
Conformation of compounds **1h** (yellow) and **1l** (green) in BChE.

The conformation of **1l** (together with **1h**, being two of the most potent inhibitors of BChE in all series) is shown in Figure 3. The acylhydrazine-1-carboxamide linker is in close proximity to the catalytic active site, able to form H-bond interactions with amino acid residues of the catalytic triad. The tridecyl moiety of both ligands was buried deeper in the cavity of BChE when compared to AChE surrounded by hydrophobic amino acids Phe329 and Ala328. This corresponded to the general assumption that the cavity of BChE is more spacious than that of AChE. Nevertheless, both enzymes were capable of accommodateing the tridecyl moiety without any difficulties.

## 3. Materials and Methods

### 3.1. Chemistry

#### 3.1.1. General

All chemicals were purchased from Merck KGaA (Darmstadt, Germany), Acros Organics B.V.B.A. (Geel, Belgium), Penta Chemicals Unlimited (Prague, Czech Republic) and Avantor (Stříbrná Skalice, Czech Republic) and were used as received. The progress of reactions was monitored via thin-layer chromatography (TLC). Plates were coated with 0.2 mm Merck 60 F254 silica gel (Merck, Darmstadt, Germany) and were visualized via UV light (254 and 366 nm). Alternatively, ALUGRAM SIL G/UV_254_ aluminum plates (Macherey-Nagel GmbH, Düren, Germany) coated with a 0.2 mm silica gel layer (60A, with a fluorescent indicator for 254 nm) were used (method C). Avantor Silica Gel with a particle size of 40–60 µm was used for column chromatography without prior activation. Melting points were recorded using a Büchi B-560 apparatus (BÜCHI Labortechnik AG, Flawil, Switzerland), and they were without correction.

The structures of both known and new compounds were determined via ^1^H NMR and ^13^C NMR spectroscopy. NMR spectra were measured in a dimethyl sulfoxide (DMSO-*d_6_*) solution at an ambient temperature with a JNM-ECZ 600R (600 MHz for ^1^H and 151 MHz for ^13^C; JEOL, Tokio, Japan) or a Varian VNMR S500 instrument (500 MHz for ^1^H and 126 MHz for ^13^C; Varian Comp. Palo Alto, CA, USA). The chemical shifts (δ) are reported in parts per million (ppm) and are indirectly related to tetramethylsilane via residual signals of the solvent (2.50 for ^1^H and 39.51 for ^13^C spectra). Coupling constants (*J*) are given in Hz. Infrared spectra were recorded via a Nicolet 6700 FT-IR spectrometer (Thermo Fisher Scientific, Waltham, MA, USA) in the range of 600–4000 cm^−1^. The attenuated total reflectance technique (ATR) on a germanium crystal was used. Elemental analysis was performed on a Vario MICRO Cube Elemental Analyzer (Elementar Analysensysteme, Hanau, Germany). Calculated and found values are given as percentages; all the calculated and experimental values were within ±0.4%.

Lipophilicity (expressed as consensus log *P*_o/w_) was determined in silico using a freely available tool SwissADME (http://www.swissadme.ch/index.php, accessed 5 December 2022).

#### 3.1.2. Synthesis

##### Synthesis of Parent Hydrazides

Hydrazide precursors for final compounds **1–3** were obtained commercially or prepared in-house. Their synthesis involved the hydrazinolysis of methyl esters in boiling ethanol. The esters were prepared from commercially available acyl chlorides and methanol in the presence of an excess of triethylamine. Alternatively, free acid was esterified with methanol using a catalytic amount of sulfuric acid under reflux.

We previously reported on the preparation of 4-iodobenzohydrazide [15], and we also used the procedure for 2-phenylacetohydrazide [16], [1,1’-biphenyl]-4-carbohydrazide [17], 4-phenoxybenzohydrazide [18], 4-(trifluoromethoxy)benzohydrazide [19], 3-(trifluoromethyl)benzohydrazide [20], 2-(trifluoromethyl)benzohydrazide [21], 4-(hydrazinecarbonyl)benzenesulfonamide [22], 4-cyanobenzohydrazide [23] and 4-(trifluoromethyl)benzenesulfonylhydrazide [24].

The spectral and physical characterizations (NMR, IR and melting points) of these precursor compounds were in accordance with literature. Their purity was checked via elemental analysis.

##### Synthesis of Hydrazine-1-carboxamides

Method A

Substituted benzohydrazide (1.0 mmol) was dissolved in acetonitrile (5 mL) and mixed with *N*,*N*-diisopropylethylamine (DIPEA; 2.0 mmol, 348 µL) followed by *N*-succinimidyl *N*-methylcarbamate (1.5 mmol; 258.2 mg). The reaction mixture was stirred at room temperature for 24 h, the formed precipitate was filtered off, it was washed with cold water and diethyl ether, and it was crystallized from ethyl acetate.

Method B

Triphosgene (0.4 mol, 118.7 mg) was dissolved in anhydrous dichloromethane (DCM; 5 mL) under a nitrogen atmosphere. Then, tridecylamine (1.01 mmol; 201.4 mg) dissolved in anhydrous DCM (5 mL) was added dropwise. After 30 min of stirring at room temperature, triethylamine (2.1 mmol, 293 µL) was added. After an additional 30 min, substituted benzohydrazide (1.0 mmol) was added. The reaction mixture was stirred for 10 h at room temperature and was then evaporated to dryness, treated with water (10 mL) and extracted with ethyl acetate (3 × 15 mL). The combined organic phase was dried over anhydrous sodium sulfate, filtered off and evaporated to dryness to give the final product, which was crystallized from ethyl acetate.

Method C

Both alkyl isocyanates were prepared via the Curtius rearrangement from commercially available acyl chlorides according to the known method [10]. The crude isocyanate was used immediately for a subsequent reaction.

Then, 1 mmol of the appropriate benzohydrazide (benzenesulfonyl hydrazide) was dissolved/suspended in 10 mL of anhydrous acetonitrile and was heated to a boil under a nitrogen atmosphere, and then a 1.1 equivalent (1.1 mmol, as a solution in toluene) of alkyl isocyanate was added in one portion to the vigorously stirred reaction mixture. The reaction mixture was heated under reflux for one hour. The solvent was evaporated under reduced pressure. The crude product was purified via column chromatography on silica gel using gradient elution (pure DCM **→** mixture of DCM to CH_3_OH; 93:7 ^V^/_V_). The reaction progress was monitored via TLC (DCM + CH_3_OH; 93:7 ^V^/_V_).

*N*-Methyl-2-[4-(trifluoromethoxy)benzoyl]hydrazine-1-carboxamide **1a**. White solid. Yield 91% (method C), mp 237–239 °C. ^1^H NMR (600 MHz, DMSO-*D*_6_) *δ* 10.18 (1H, s, NH), 8.00–7.96 (2H, m, H2, H6), 7.88 (1H, s, NH), 7.46–7.43 (2H, m, H3, H5), 6.42 (1H, q, *J* = 4.5 Hz, NH-CH_3_), 2.54 (3H, d, *J* = 4.6 Hz, NH-CH_3_). ^13^C NMR (151 MHz, DMSO-*D*_6_) *δ* 165.75, 159.36, 151.08, 132.46, 130.50, 121.14, 120.91 (q, *J* = 255.0 Hz), 26.75. IR (ATR): 636, 760, 851, 912, 930, 988, 1016, 1064, 1165, 1211, 1252, 1344, 1424, 1502, 1544, 1584, 1609, 1644, 2890, 3074, 3274 cm^−1^. Elemental analysis for C_10_H_10_F_3_N_3_O_3_ (277.20); calculated: C, 43.33; H, 3.64; N, 15.16, found: C, 43.44; H, 3.77; N, 15.01. R*_f_*: 0.46.

*N*-Tridecyl-2-[4-(trifluoromethoxy)benzoyl]hydrazine-1-carboxamide **1b**. White needle-like solid. Yield 90% (method C), mp 200–201 °C. ^1^H NMR (600 MHz, DMSO-*D*_6_) *δ* 9.98 (1H, s, NH), 7.97 (2H, d, *J* = 8.3 Hz, H2, H6), 7.63 (1H, s, NH), 7.39 (2H, d, *J* = 8.3 Hz, H3, H5), 6.24 (1H, t, *J* = 5.9 Hz, NH-CH_2_), 3.01 (2H, q, *J* = 6.6 Hz, NH-CH_2_), 1.38 (2H, p, *J* = 6.9 Hz, C^2^H_2_), 1.24–1.21 (20H, m, C^3^H_2_, C^4^H_2_, C^5^H_2_, C^6^H_2_, C^7^H_2_, C^8^H_2_, C^9^H_2_, C^10^H_2_, C^11^H_2_, C^12^H_2_), 0.83 (3H, t, *J* = 6.9 Hz, CH_3_). ^13^C NMR (151 MHz, DMSO-*D*_6_) *δ* 165.68, 158.66, 151.17, 132.65, 130.38, 121.43, 120.91 (q, *J* = 255.0 Hz), 31.75, 30.32, 29.50, 29.49, 29.48, 29.47, 29.46, 29.44, 29.29, 29.12, 26.84, 22.49, 14.25. IR (ATR): 627, 722, 862, 899, 922, 1015, 1059, 1168, 1204, 1266, 1339, 1470, 1490, 1549, 1591, 1640, 1668, 2853, 2921, 3278 cm^−1^. Elemental analysis for C_22_H_34_F_3_N_3_O_3_ (445.53); calculated: C, 59.31; H, 7.69; N, 9.43, found: C, 59.45; H, 7.61; N, 9.50. R*_f_*: 0.46.

2-(4-Fluorobenzoyl)-*N*-methylhydrazine-1-carboxamide **1c**. White solid. Yield 33% (method A), mp 221–223 °C. ^1^H NMR (500 MHz, DMSO-*D*_6_) *δ* 10.13 (1H, s, NH), 7.99–7.93 (2H, m, H2, H6), 7.86 (1H, s, NH), 7.34–7.27 (2H, m, H3, H5), 6.43 (1H, q, *J* = 4.9 Hz, NH-CH_3_), 2.57 (3H, d, *J* = 4.6 Hz, NH-CH_3_). ^13^C NMR (126 MHz, DMSO-*D*_6_) *δ* 165.56, 164.29 (d, *J* = 249.0 Hz), 159.08, 130.46 (d, *J* = 9.1 Hz), 129.46 (d, *J* = 2.9 Hz), 115.42 (d, *J* = 21.8 Hz), 26.42. IR (ATR): 3309, 3070, 1665, 1644, 1603, 1581, 1542, 1494, 1341, 1299, 1254, 1230, 1163, 1100, 1010, 912, 848, 927, 813, 758, 650, 629 cm^−1^. Elemental analysis for C_9_H_10_FN_3_O_2_ (211.20); calculated: C, 51.18; H, 4.77; N, 19.90, found: C, 51.24; H, 4.70; N, 20.01. 

2-(4-Fluorobenzoyl)-*N*-tridecylhydrazine-1-carboxamide **1d**. White solid. Yield 25% (method B), mp 196–198 °C. ^1^H NMR (600 MHz, DMSO-*D*_6_) *δ* 9.95 (1H, d, *J* = 2.0 Hz, NH), 7.94–7.90 (2H, m, H2, H6), 7.62 (1H, s, NH), 7.26–7.21 (2H, m, H3, H5), 6.26 (1H, t, *J* = 5.9 Hz, NH-CH_2_), 3.00 (2H, q, *J* = 6.6 Hz, NH-CH_2_), 1.38 (2H, p, *J* = 7.0 Hz, C^2^H_2_), 1.29–1.21 (20H, m, C^3^H_2_, C^4^H_2_, C^5^H_2_, C^6^H_2_, C^7^H_2_, C^8^H_2_, C^9^H_2_, C^10^H_2_, C^11^H_2_, C^12^H_2_), 0.83 (3H, t, *J* = 7.0 Hz, CH_3_). ^13^C NMR (151 MHz, DMSO-*D*_6_) *δ* 165.85, 164.67 (d, *J* = 249.1 Hz), 158.76, 130.72 (d, *J* = 9.1 Hz), 130.01, 115.66 (d, *J* = 21.9 Hz), 39.87, 31.77, 30.35, 29.52, 29.50, 29.48, 29.31, 29.15, 26.84, 22.52, 14.31. IR (ATR): 3303, 3073, 2922, 2850, 1660, 1638, 1605, 1568, 1501, 1465, 1339, 1300, 1279, 1258, 1232, 1164, 1068, 914, 849, 771, 755, 725, 642, 627 cm^−1^. Elemental analysis for C_21_H_34_FN_3_O_2_ (379.52); calculated: C, 66.46; H, 9.03; N, 11.07, found: C, 66.41; H, 9.10; N, 11.05. 

2-(4-Chlorobenzoyl)-*N*-methylhydrazine-1-carboxamide **1e**. White solid. Yield 59% (method A), mp 227–229 °C. ^1^H NMR (500 MHz, DMSO-*D*_6_) *δ* 10.18 (1H, s, NH), 7.92–7.88 (3H, m, NH, H2, H6), 7.57–7.54 (2H, m, H3, H5), 6.44 (1H, q, *J* = 4.7 Hz, NH-CH_3_), 2.57 (3H, d, *J* = 4.6 Hz, NH-CH_3_). ^13^C NMR (126 MHz, DMSO-*D*_6_) *δ* 165.61, 159.02, 136.64, 131.74, 129.70, 128.56, 26.42. IR (ATR): 3318, 3253, 3063, 1663, 1642, 1597, 1574, 1543, 1487, 1424, 1340, 1252, 1095, 1010, 910, 895, 845, 759, 727, 700, 663, 651, 625, 608 cm^−1^. Elemental analysis for C_9_H_10_ClN_3_O_2_ (227.65); calculated: C, 47.49; H, 4.43; N, 18.46, found: C, 47.54; H, 4.40; N, 18.51. 

2-(4-Chlorobenzoyl)-*N*-tridecylhydrazine-1-carboxamide **1f**. White solid. Yield 26% (method B), mp 195–197 °C. ^1^H NMR (500 MHz, DMSO-*D*_6_) *δ* 10.16 (1H, s, NH), 7.91–7.87 (2H, m, H2, H6), 7.80 (1H, s, NH), 7.56–7.53 (2H, m, H3, H5), 6.49 (1H, t, *J* = 6.0 Hz, NH-CH_2_), 2.99 (2H, q, *J* = 6.5 Hz, NH-CH_2_), 1.36 (2H, p, *J* = 6.9 Hz, C^2^H_2_), 1.23–1.15 (20H, m, C^3^H_2_, C^4^H_2_, C^5^H_2_, C^6^H_2_, C^7^H_2_, C^8^H_2_, C^9^H_2_, C^10^H_2_, C^11^H_2_, C^12^H_2_), 0.84 (3H, t, *J* = 6.8 Hz, CH_3_). ^13^C NMR (126 MHz, DMSO-*D*_6_) *δ* 165.54, 158.45, 136.64, 131.75, 129.67, 128.57, 39.41, 31.49, 30.04, 29.26, 29.21, 29.12, 29.03, 28.91, 27.13, 26.50, 22.29, 14.14. IR (ATR): 3309, 2952, 2919, 2850, 1663, 1637, 1599, 1580, 1540, 1486, 1469, 1338, 1279, 1261, 1092, 1012, 907, 844, 751, 720, 667, 636, 622, 606 cm^−1^. Elemental analysis for C_21_H_34_ClN_3_O_2_ (395.97); calculated: C, 63.70; H, 8.66; N, 10.61, found: C, 63.74; H, 8.50; N, 10.51. 

2-(4-Bromobenzoyl)-*N*-methylhydrazine-1-carboxamide **1g**. White solid. Yield 40% (method A), mp 225–227 °C. ^1^H NMR (500 MHz, DMSO-*D*_6_) *δ* 10.18 (1H, s, NH), 7.89 (1H, s, NH), 7.84–7.81 (2H, m, H2, H6), 7.71–7.68 (2H, m, H3, H5), 6.44 (1H, q, *J* = 5.0 Hz, NH-CH_3_), 2.57 (3H, d, *J* = 4.5 Hz, NH-CH_3_). ^13^C NMR (126 MHz, DMSO-*D*_6_) *δ* 165.74, 159.00, 132.11, 131.50, 129.88, 125.58, 26.43. IR (ATR): 3287, 1683, 1665, 1637, 1589, 1558, 1536, 1482, 1419, 1342, 1315, 1277, 1254, 1176, 1108, 1072, 1008, 898, 847, 824, 759, 715 cm^−1^. Elemental analysis for C_9_H_10_BrN_3_O_2_ (272.10); calculated: C, 39.73; H, 3.70; N, 15.44, found: C, 39.74; H, 3.65; N, 15.51. 

2-(4-Bromobenzoyl)-*N*-tridecylhydrazine-1-carboxamide **1h**. White solid. Yield 34% (method B), mp 144–146 °C. ^1^H NMR (500 MHz, DMSO-*D*_6_) *δ* 10.16 (1H, s, NH), 7.86–7.81 (3H, m, NH, H2, H6), 7.72–7.68 (2H, m, H3, H5), 6.49 (1H, t, *J* = 5.0 Hz, NH-CH_2_), 3.06 (2H, q, *J* = 6.5 Hz, NH-CH_2_), 1.36 (2H, p, *J* = 6.8 Hz, C^2^H_2_), 1.24–1.19 (20H, m, C^3^H_2_, C^4^H_2_, C^5^H_2_, C^6^H_2_, C^7^H_2_, C^8^H_2_, C^9^H_2_, C^10^H_2_, C^11^H_2_, C^12^H_2_), 0.84 (3H, t, *J* = 6.8 Hz, CH_3_). ^13^C NMR (126 MHz, DMSO-*D*_6_) *δ* 165.59, 158.38, 132.48, 131.47, 129.82, 127.34, 38.92, 31.46, 29.23, 29.19, 29.10, 29.00, 28.88, 28.69, 27.12, 26.48, 25.98, 22.26, 14.11. IR (ATR): 2954, 2919, 2849, 1663, 1637, 1595, 1521, 1496, 1468, 1398, 1377, 1340, 1263, 1147, 1121, 1074, 1010, 981, 943, 897, 841, 754, 721, 646, 632, 625, 612 cm^−1^. Elemental analysis for C_21_H_34_BrN_3_O_2_ (440.43); calculated: C, 57.27; H, 7.78; N, 9.54, found: C, 57.24; H, 7.75; N, 9.59. 

2-(4-Iodobenzoyl)-*N*-methylhydrazine-1-carboxamide **1i**. White solid. Yield 24% (method A), mp 238–240 °C. ^1^H NMR (500 MHz, DMSO-*D*_6_) *δ* 10.16 (1H, s, NH), 7.89–7.83 (3H, m, NH, H2, H6), 7.70–7.64 (2H, m, H3, H5), 6.43 (1H, s, NH-CH_3_), 2.57 (3H, s, NH-CH_3_). ^13^C NMR (126 MHz, DMSO-*D*_6_) *δ* 165.99, 158.99, 137.35, 132.41, 129.69, 99.51, 26.43. IR (ATR): 3286, 1664, 1638, 1586, 1558, 1538, 1480, 1418, 1343, 1310, 1276, 1253, 1173, 1107, 1064, 1005, 898, 845, 825, 754, 706, 651, 633 cm^−1^. Elemental analysis for C_9_H_10_IN_3_O_2_ (319.10); calculated: C, 33.88; H, 3.16; N, 13.17, found: C, 33.79; H, 3.15; N, 13.10. 

2-(4-Iodobenzoyl)-*N*-tridecylhydrazine-1-carboxamide **1j**. White solid. Yield 24% (method B), mp 190–193 °C. ^1^H NMR (600 MHz, DMSO-*D*_6_) *δ* 10.12 (1H, s, NH), 7.90–7.83 (3H, m, NH, H2, H6), 7.52 (2H, d, *J* = 8.3 Hz, H3, H5), 6.47 (1H, t, *J* = 5.0 Hz, NH-CH_2_), 2.90 (2H, q, *J* = 6.6 Hz, NH-CH_2_), 1.31 (2H, p, *J* = 6.8 Hz, C^2^H_2_), 1.25–1.19 (20H, m, C^3^H_2_, C^4^H_2_, C^5^H_2_, C^6^H_2_, C^7^H_2_, C^8^H_2_, C^9^H_2_, C^10^H_2_, C^11^H_2_, C^12^H_2_), 0.81 (3H, t, *J* = 6.9 Hz, CH_3_). ^13^C NMR (151 MHz, DMSO-*D*_6_) *δ* 157.18, 154.85, 138.64, 127.46, 124.00, 99.23, 40.76, 31.83, 29.95, 29.59, 29.57, 29.55, 29.52, 29.27, 29.25, 29.07, 26.76, 22.63, 14.46. IR (ATR): 3340, 2956, 2919, 2850, 1691, 1613, 1583, 1536, 1479, 1463, 1398, 1351, 1332, 1284, 1263, 1247, 1198, 1144, 1076, 1033, 1006, 967, 938, 823, 720, 668, 639, 627, 612 cm^−1^. Elemental analysis for C_21_H_34_IN_3_O_2_ (487.43); calculated: C, 51.75; H, 7.03; N, 8.62, found: C, 51.84; H, 7.05; N, 8.59. 

*N*-Methyl-2-(4-nitrobenzoyl)hydrazine-1-carboxamide **1k**. Yellowish solid. Yield 39% (method A), mp 252–254 °C. ^1^H NMR (500 MHz, DMSO-*D*_6_) *δ* 10.44 (1H, s, NH), 8.33 (2H, d, *J* = 8.7 Hz, H3, H5), 8.15–8.00 (3H, m, NH, H2, H6), 6.52 (1H, s, NH-CH_3_), 2.59 (3H, s, NH-CH_3_). ^13^C NMR (126 MHz, DMSO-*D*_6_) *δ* 165.11, 158.88, 149.47, 138.73, 129.33, 123.67, 26.45. IR (ATR): 3283, 3070, 1670, 1635, 1604, 1582, 1547, 1519, 1490, 1419, 1341, 1318, 1258, 1190, 1171, 1107, 1014, 989, 904, 875, 850, 716, 680, 644, 609 cm^−1^. Elemental analysis for C_9_H_10_N_4_O_4_ (238.20); calculated: C, 45.38; H, 4.23; N, 23.52, found: C, 45.49; H, 4.15; N, 23.60. 

2-(4-Nitrobenzoyl)-*N*-tridecylhydrazine-1-carboxamide **1l**. Yellowish solid. Yield 30% (method B), mp 252–252 °C. ^1^H NMR (500 MHz, DMSO-*D*_6_) *δ* 10.45 (1H, s, NH), 8.31 (2H, d, *J* = 8.8 Hz, H3, H5), 8.16–8.05 (3H, m, NH, H2, H6), 6.48 (1H, t, *J* = 5.0 Hz, NH-CH_2_), 2.71 (2H, q, *J* = 6.6 Hz, NH-CH_2_), 1.53 (2H, p, *J* = 6.8 Hz, C^2^H_2_), 1.24–1.19 (20H, m, C^3^H_2_, C^4^H_2_, C^5^H_2_, C^6^H_2_, C^7^H_2_, C^8^H_2_, C^9^H_2_, C^10^H_2_, C^11^H_2_, C^12^H_2_), 0.83 (3H, t, *J* = 7.0 Hz, CH_3_). ^13^C NMR (126 MHz, DMSO-*D*_6_) *δ* 164.33, 159.12, 150.04, 136.25, 129.64, 124.02, 38.90, 31.52, 29.29, 29.26, 29.24, 29.16, 29.07, 28.94, 28.77, 27.14, 26.08, 22.32, 14.18. IR (ATR): 3268, 2954, 2929, 2850, 1673, 1605, 1589, 1530, 1481, 1344, 1326, 1286, 1191, 1173, 1147, 1118, 1039, 1009, 882, 845, 717, 648, 624, 606 cm^−1^. Elemental analysis for C_21_H_34_N_4_O_4_ (406.53); calculated: C, 62.05; H, 8.43; N, 13.78, found: C, 62.04; H, 8.35; N, 15.69. 

2-(4-Cyanobenzoyl)-*N*-methylhydrazine-1-carboxamide **1m**. White solid. Yield 94% (method C), mp 209–210 °C (decomp.). ^1^H NMR (600 MHz, DMSO-*D*_6_) *δ* 10.31 (1H, s, NH), 8.01–7.98 (2H, m, H2, H6), 7.95–7.92 (3H, m, NH, H3, H5), 6.45 (1H, q, *J* = 4.4 Hz, NH-CH_3_), 2.54 (3H, d, *J* = 4.5 Hz, NH-CH_3_). ^13^C NMR (151 MHz, DMSO-*D*_6_) *δ* 165.62, 159.22, 137.38, 132.93, 128.97, 118.83, 114.52, 26.77. IR (ATR): 612, 760, 774, 859, 905, 991, 1018, 1172, 1259, 1312, 1338, 1424, 1496, 1541, 1586, 1641, 1669, 2230, 3297 cm^−1^. Elemental analysis for C_10_H_10_N_4_O_2_ (218.22); calculated: C, 55.04; H, 4.62; N, 25.68, found: C, 55.18; H, 4.74; N, 25.59. R*_f_*: 0.27.

2-(4-Cyanobenzoyl)-*N*-tridecylhydrazine-1-carboxamide **1n**. White solid. Yield 99% (method C), mp 206–208°C. ^1^H NMR (600 MHz, DMSO-*D*_6_) *δ* 10.22 (1H, s, NH), 7.99 (2H, d, *J* = 8.4 Hz, H2, H6), 7.92 (2H, d, *J* = 8.1 Hz, H3, H5), 7.76 (1H, s, NH), 6.37 (1H, t, *J* = 5.8 Hz, NH-CH_2_), 2.99 (2H, q, *J* = 6.6 Hz, NH-CH_2_), 1.36 (2H, p, *J* = 6.9 Hz, C^2^H_2_), 1.21–1.12 (20H, m, C^3^H_2_, C^4^H_2_, C^5^H_2_, C^6^H_2_, C^7^H_2_, C^8^H_2_, C^9^H_2_, C^10^H_2_, C^11^H_2_, C^12^H_2_), 0.82 (3H, t, *J* = 7.0 Hz, CH_3_). ^13^C NMR (151 MHz, DMSO-*D*_6_) *δ* 165.51, 158.57, 137.50, 132.86, 128.92, 118.74, 114.54, 31.79, 30.34, 29.56, 29.55, 29.54, 29.52, 29.51, 29.50, 29.32, 29.18, 26.83, 22.56, 14.38. IR (ATR): 626, 650, 724, 757, 855, 912, 1016, 1060, 1245, 1258, 1339, 1465, 1489, 1544, 1586, 1638, 1667, 2851, 2922, 3271, 3307 cm^−1^. Elemental analysis for C_22_H_34_N_4_O_2_ (386.54); calculated: C, 68.36; H, 8.87; N, 14.49, found: C, 68.42; H, 8.99; N, 14.40. R*_f_*: 0.45.

*N*-Methyl-2-(4-methylbenzoyl)hydrazine-1-carboxamide **1o**. White solid. Yield 36% (method A), mp 193–195 °C. ^1^H NMR (500 MHz, DMSO-*D*_6_) *δ* 10.01 (1H, s, NH), 7.83–7.87 (3H, m, NH, H2, H6), 7.27 (2H, d, *J* = 8.1 Hz, H3, H5), 6.38 (1H, q, *J* = 5.7 Hz, NH-CH_3_), 2.57 (3H, d, *J* = 4.6 Hz, NH-CH_3_), 2.35 (3H, s, Ph-CH_3_). ^13^C NMR (126 MHz, DMSO-*D*_6_) *δ* 166.49, 159.17, 144.27, 130.16, 128.96, 127.77, 26.44, 21.18. IR (ATR): 3310, 3254, 3063, 1666, 1644, 1613, 1575, 1541, 1505, 1420, 1337, 1255, 1191, 1126, 1108, 1016, 910, 840, 750, 701, 646, 634, 629 cm^−1^. Elemental analysis for C_10_H_13_N_3_O_2_ (207.23); calculated: C, 57.96; H, 6.32; N, 20.28, found: C, 57.99; H, 6.35; N, 20.30. 

2-(4-Methylbenzoyl)-*N*-tridecylhydrazine-1-carboxamide **1p**. White solid. Yield 22% (method B), mp 159–161 °C. ^1^H NMR (500 MHz, DMSO-*D*_6_) *δ* 10.51 (1H, s, NH), 7.87–7.83 (3H, m, NH, H2, H6), 7.28–7.24 (2H, m, H3, H5), 6.48 (1H, t, *J* = 5.0 Hz, NH-CH_2_), 3.04 (2H, q, *J* = 6.6 Hz, NH-CH_2_), 2.36 (3H, s, Ph-CH_3_), 1.36 (2H, p, *J* = 6.8 Hz, C^2^H_2_), 1.26–1.19 (20H, m, C^3^H_2_, C^4^H_2_, C^5^H_2_, C^6^H_2_, C^7^H_2_, C^8^H_2_, C^9^H_2_, C^10^H_2_, C^11^H_2_, C^12^H_2_), 0.84 (3H, t, *J* = 7.0 Hz, CH_3_). ^13^C NMR (126 MHz, DMSO-*D*_6_) *δ* 166.35, 158.53, 141.66, 130.15, 128.93, 127.71, 39.10, 31.47, 30.03, 29.28, 29.24, 29.20, 29.17, 29.01, 28.88, 26.48, 22.26, 21.15, 14.11. IR (ATR): 3293, 2917, 2850, 1666, 1643, 1614, 1586, 1542, 1504, 1468, 1377, 1339, 1262, 1190, 1124, 1063, 1017, 912, 838, 746, 721, 655, 641, 625 cm^−1^. Elemental analysis for C_22_H_37_N_3_O_2_ (375.56); calculated: C, 70.36; H, 9.93; N, 11.19, found: C, 70.44; H, 10.00; N, 11.15. 

2-[(4-(*tert*-Butyl)benzoyl]-*N*-methylhydrazine-1-carboxamide **1q**. White solid. Yield 25% (method A), mp 180–182 °C. ^1^H NMR (500 MHz, DMSO-*D*_6_) *δ* 10.02 (1H, s, NH), 7.85–7.81 (3H, m, NH, H2, H6), 7.50–7.47 (2H, m, H3, H5), 6.37 (1H, q, *J* = 4.7 Hz, NH-CH_3_), 2.57 (3H, d, *J* = 4.6 Hz, NH-CH_3_), 1.29 (9H, s, C-CH_3_). ^13^C NMR (126 MHz, DMSO-*D*_6_) *δ* 166.45, 159.19, 154.68, 130.20, 127.64, 125.22, 34.86, 31.13, 26.46. IR (ATR): 3353, 3221, 2963, 2906, 2870, 1682, 1655, 1611, 1573, 1519, 1463, 1408, 1366, 1297, 1286, 1196, 1163, 1113, 1079, 1019, 926, 890, 854, 839, 765, 705, 658, 623, 607 cm^−1^. Elemental analysis for C_13_H_19_N_3_O_2_ (249.31); calculated: C, 62.63; H, 7.68; N, 16.85, found: C, 62.59; H, 7.65; N, 16.90. 

2-[(4-(*tert*-Butyl)benzoyl]-*N*-tridecylhydrazine-1-carboxamide **1r**. White solid. Yield 39% (method B), mp 103–105 °C. ^1^H NMR (500 MHz, DMSO-*D*_6_) *δ* 9.99 (1H, s, NH), 7.80–7.77 (2H, m, H2, H6), 7.75 (1H, s, NH), 7.48–7.45 (2H, m, H3, H5), 6.44 (1H, t, *J* = 5.8 Hz, NH-CH_2_), 2.98 (2H, q, *J* = 6.6 Hz, NH-CH_2_), 1.34 (2H, p, *J* = 6.6 Hz, C^2^H_2_), 1.28–1.19 (29H, m, C-CH_3_, C^3^H_2_, C^4^H_2_, C^5^H_2_, C^6^H_2_, C^7^H_2_, C^8^H_2_, C^9^H_2_, C^10^H_2_, C^11^H_2_, C^12^H_2_), 0.82 (3H, t, *J* = 6.9 Hz, CH_3_). ^13^C NMR (126 MHz, DMSO-*D*_6_) *δ* 166.99, 159.05, 155.17, 130.32, 127.90, 125.59, 39.96, 31.76, 31.38, 29.51, 29.48, 29.46, 29.39, 29.27, 29.16, 28.98, 27.41, 26.73, 26.25, 22.57, 14.43. IR (ATR): 3356, 3258, 2955, 2920, 2851, 1702, 1628, 1610, 1544, 1500, 1468, 1364, 1333, 1268, 1258, 1240, 1123, 1016, 978, 895, 852, 788, 761, 721, 663, 652, 635, 616 cm^−1^. Elemental analysis for C_25_H_43_N_3_O_2_ (417.64); calculated: C, 71.90; H, 10.38; N, 10.06, found: C, 72.01; H, 10.40; N, 10.13. 

*N*-Methyl-2-(4-phenylbenzoyl)hydrazine-1-carboxamide **1s**. White solid. Yield 20% (method A), mp 172–174 °C. ^1^H NMR (600 MHz, DMSO-*D*_6_) *δ* 10.12 (1H, s, NH), 7.96 (2H, d, *J* = 8.2 Hz, H2, H6), 7.85 (1H, s, NH), 7.75 (2H, d, *J* = 8.3 Hz, H3, H5), 7.70 (2H, d, *J* = 7.7 Hz, H2’, H6’), 7.46 (2H, t, *J* = 7.5 Hz, H3’, H5’), 7.37 (1H, t, *J* = 7.3 Hz, H4’), 6.40 (1H, q, *J* = 5.7 Hz, NH-CH_3_), 2.55 (3H, d, *J* = 4.6 Hz, NH-CH_3_). ^13^C NMR (151 MHz, DMSO-*D*_6_) *δ* 166.61, 159.49, 143.66, 139.65, 132.14, 129.59, 128.82, 128.66, 127.42, 126.99, 26.81. IR (ATR): 3411, 3209, 1706, 1680, 1650, 1637, 1609, 1561, 1485, 1413, 1329, 1240, 1207, 1130, 1105, 1006, 901, 857, 780, 744, 690, 661, 636, 609 cm^−1^. Elemental analysis for C_15_H_15_N_3_O_2_ (269.30); calculated: C, 66.90; H, 5.61; N, 15.60, found: C, 66.96; H, 5.55; N, 15.70. 

2-(4-Phenylbenzoyl)-*N*-tridecylhydrazine-1-carboxamide **1t**. White solid. Yield 84% (method B), mp 249–251 °C. ^1^H NMR (600 MHz, DMSO-*D*_6_) *δ* 10.63 (1H, s, NH), 8.02 (2H, d, *J* = 8.2 Hz, H2, H6), 7.97 (1H, s, NH), 7.81 (2H, d, *J* = 8.2 Hz, H3, H5), 7.72 (2H, d, *J* = 7.8 Hz, H2’, H6’), 7.47 (2H, t, *J* = 7.7 Hz, H3’, H5’), 7.39 (1H, t, *J* = 7.5 Hz, H4’), 6.46 (1H, t, *J* = 5.0 Hz, NH-CH_2_), 2.69 (2H, q, *J* = 6.3 Hz, NH-CH_2_), 1.40 (2H, p, *J* = 6.8 Hz, C^2^H_2_), 1.24–1.17 (20H, m, C^3^H_2_, C^4^H_2_, C^5^H_2_, C^6^H_2_, C^7^H_2_, C^8^H_2_, C^9^H_2_, C^10^H_2_, C^11^H_2_, C^12^H_2_), 0.81 (3H, t, *J* = 6.9 Hz, CH_3_). ^13^C NMR (151 MHz, DMSO-*D*_6_) *δ* 166.06, 158.32, 144.77, 139.36, 129.74, 129.64, 129.02, 128.92, 127.51, 127.40, 39.24, 31.82, 29.59, 29.56, 29.54, 29.46, 29.36, 29.24, 29.07, 27.46, 26.39, 22.62, 14.48. IR (ATR): 2955, 2920, 2850, 1673, 1610, 1575, 1558, 1520, 1484, 1468, 1449, 1401, 1303, 1238, 1163, 1146, 1078, 1036, 1006, 852, 779, 742, 722, 698, 648, 631, 614 cm^−1^. Elemental analysis for C_27_H_39_N_3_O_2_ (437.63); calculated: C, 74.10; H, 8.98; N, 9.60, found: C, 74.17; H, 9.05; N, 9.55. 

2-(4-Methoxybenzoyl)-*N*-methylhydrazine-1-carboxamide **1u**. White solid. Yield 34% (method A), mp 171–173 °C. ^1^H NMR (500 MHz, DMSO-*D*_6_) *δ* 9.95 (1H, s, NH), 7.88–7.85 (2H, m, H2, H6), 7.78 (1H, s, NH), 7.02–6.99 (2H, m, H3, H5), 6.37 (1H, q, *J* = 5.0 Hz, NH-CH_3_), 3.81 (3H, s, OCH_3_), 2.57 (3H, d, *J* = 4.6 Hz, NH-CH_3_). ^13^C NMR (126 MHz, DMSO-*D*_6_) *δ* 166.10, 162.05, 159.24, 129.63, 125.12, 113.67, 55.55, 26.43. IR (ATR): 3347, 3323, 3004, 2944, 1685, 1634, 1610, 1574, 1524, 1496, 1467, 1420, 1324, 1260, 1173, 1104, 1027, 980, 902, 840, 797, 767, 738, 700, 652, 624 cm^−1^. Elemental analysis for C_10_H_13_N_3_O_3_ (223.23); calculated: C, 53.81; H, 5.87; N, 18.82, found: C, 53.89; H, 5.85; N, 18.90. 

2-(4-Methoxybenzoyl)-*N*-tridecylhydrazine-1-carboxamide **1v**. White solid. Yield 27% (method B), mp 149–151 °C. ^1^H NMR (500 MHz, DMSO-*D*_6_) *δ* 9.96 (1H, s, NH), 7.86 (2H, d, *J* = 8.9 Hz, H2, H6), 7.76 (1H, s, NH), 6.99 (2H, d, *J* = 8.9 Hz, H3, H5), 6.51 (1H, t, *J* = 5.0 Hz, NH-CH_2_), 3.80 (3H, s, OCH_3_), 2.98 (2H, q, *J* = 6.6 Hz, NH-CH_2_), 1.35 (2H, p, *J* = 6.8 Hz, C^2^H_2_), 1.25–1.19 (20H, m, C^3^H_2_, C^4^H_2_, C^5^H_2_, C^6^H_2_, C^7^H_2_, C^8^H_2_, C^9^H_2_, C^10^H_2_, C^11^H_2_, C^12^H_2_), 0.84 (3H, t, *J* = 6.9 Hz, CH_3_). ^13^C NMR (126 MHz, DMSO-*D*_6_) *δ* 165.99, 162.02, 158.67, 129.60, 125.15, 113.65, 55.55, 45.41, 39.96, 31.47, 30.05, 29.24, 29.20, 29.18, 29.02, 28.88, 26.51, 22.27, 14.13. IR (ATR): 3291, 1954, 2918, 2851, 1645, 1609, 1583, 1541, 1504, 1647, 1376, 1340, 1309, 1255, 1180, 1038, 912, 842, 763, 722, 656, 640, 625 cm^−1^. Elemental analysis for C_22_H_37_N_3_O_3_ (391.56); calculated: C, 67.49; H, 9.53; N, 10.73, found: C, 67.53; H, 9.46; N, 10.65. 

*N*-Methyl-2-(4-phenoxybenzoyl)hydrazine-1-carboxamide **1w**. White solid. Yield 88% (method C), mp 176–177 °C. ^1^H NMR (600 MHz, DMSO-*D*_6_) *δ* 10.01 (1H, s, NH), 7.89–7.87 (2H, m, H2, H6), 7.80 (1H, s, NH), 7.44–7.37 (2H, m, H3’, H5’), 7.21–7.15 (1H, m, H4’), 7.07–7.03 (2H, m, H3, H5), 7.02–6.98 (2H, m, H2’, H6’), 6.36 (1H, q, *J* = 4.5 Hz, NH-CH_3_), 2.54 (3H, d, *J* = 4.5 Hz, NH-CH_3_). ^13^C NMR (151 MHz, DMSO-*D*_6_) *δ* 166.20, 160.33, 159.50, 156.11, 130.81, 130.36, 127.99, 124.90, 120.07, 117.87, 26.78. IR (ATR): 618, 658, 692, 717, 760, 873, 899, 980, 1078, 1102, 1170, 1238, 1319, 1410, 1487, 1527, 1566, 1642, 1663, 2837, 3016, 3262 cm^−1^. Elemental analysis for C_15_H_15_N_3_O_3_ (285.30); calculated: C, 63.15; H, 5.30; N, 14.73, found: C, 63.24; H, 5.20; N, 14.84. R*_f_*: 0.36.

2-(4-Phenoxybenzoyl)-*N*-tridecylhydrazine-1-carboxamide **1x**. White flakes. Yield 86% (method C), mp 127–129 °C. ^1^H NMR (600 MHz, DMSO-*D*_6_) *δ* 10.00 (1H, s, NH), 7.88 (2H, d, *J* = 8.5 Hz, H2, H6), 7.71 (1H, s, NH), 7.41 (2H, t, *J* = 7.8 Hz, H3’, H5’), 7.18 (1H, t, *J* = 7.4 Hz, H4’), 7.05 (2H, d, *J* = 7.9 Hz, H3, H5), 7.00 (2H, d, *J* = 8.6 Hz, H2’, H6’), 6.39 (1H, t, *J* = 5.9 Hz, NH-CH_2_), 2.97 (2H, q, *J* = 6.6 Hz, NH-CH_2_), 1.34 (2H, p, *J* = 7.1 Hz, C^2^H_2_), 1.22–1.18 (20H, m, C^3^H_2_, C^4^H_2_, C^5^H_2_, C^6^H_2_, C^7^H_2_, C^8^H_2_, C^9^H_2_, C^10^H_2_, C^11^H_2_, C^12^H_2_), 0.81 (3H, t, *J* = 6.8 Hz, CH_3_). ^13^C NMR (151 MHz, DMSO-*D*_6_) *δ* 166.12, 160.32, 158.91, 156.10, 130.80, 130.32, 127.98, 124.89, 120.07, 117.86, 31.83, 30.41, 29.61, 29.59, 29.58, 29.57, 29.56, 29.55, 29.38, 29.25, 26.85, 22.63, 14.48. IR (ATR): 629, 692, 721, 749, 793, 848, 872, 900, 1072, 1168, 1200, 1245, 1257, 1309, 1469, 1491, 1548, 1573, 1589, 1647, 2849, 2920, 2954, 3286 cm^−1^. Elemental analysis for C_27_H_39_N_3_O_3_ (453.63); calculated: C, 71.49; H, 8.67; N, 9.26, found: C, 71.57; H, 8.60; N, 9.12. R*_f_*: 0.48.

2-[4-(Dimethylamino)benzoyl]-*N*-methylhydrazine-1-carboxamide **1y**. White solid. Yield 31% (method A), mp 201–203 °C. ^1^H NMR (500 MHz, DMSO-*D*_6_) *δ* 9.74 (1H, s, NH), 7.78–7.73 (2H, m, H2, H6), 7.68 (1H, s, NH), 6.72–6.68 (2H, m, H3, H5), 6.32 (1H, q, *J* = 4.6 Hz, NH-CH_3_), 2.96 (6H, s, N-CH_3_), 2.56 (3H, d, *J* = 4.6 Hz, NH-CH_3_). ^13^C NMR (126 MHz, DMSO-*D*_6_) *δ* 166.57, 159.47, 152.57, 129.16, 119.38, 110.88, 39.86, 26.45. IR (ATR): 3365, 3273, 2945, 1677, 1659, 1602, 1537, 1511, 1443, 1402, 1377, 1335, 1289, 1239, 1201, 1169, 1130, 1070, 950, 917, 886, 831, 770, 703, 650 cm^−1^. Elemental analysis for C_11_H_16_N_4_O_2_ (236.28); calculated: C, 55.92; H, 6.83; N, 23.71, found: C, 55.98; H, 6.75; N, 23.80. 

2-[4-(Dimethylamino)benzoyl]-*N*-tridecylhydrazine-1-carboxamide **1z**. White solid. Yield 83% (method B), mp 148–150 °C. ^1^H NMR (500 MHz, DMSO-*D*_6_) *δ* 9.74 (1H, s, NH), 7.77–7.73 (2H, m, H2, H6), 7.62 (1H, s, NH), 6.70 (2H, d, *J* = 9.0 Hz, H3, H5), 6.37 (1H, t, *J* = 5.0 Hz, NH-CH_2_), 2.98 (2H, q, *J* = 6.5 Hz, NH-CH_2_), 2.96 (6H, s, N-CH_3_), 1.36 (2H, p, *J* = 6.9 Hz, C^2^H_2_), 1.26–1.20 (20H, m, C^3^H_2_, C^4^H_2_, C^5^H_2_, C^6^H_2_, C^7^H_2_, C^8^H_2_, C^9^H_2_, C^10^H_2_, C^11^H_2_, C^12^H_2_), 0.84 (3H, t, *J* = 6.9 Hz, CH_3_). ^13^C NMR (126 MHz, DMSO-*D*_6_) *δ* 166.43, 158.85, 152.50, 129.10, 118.26, 110.89, 39.80, 38.94, 31.50, 30.09, 29.28, 29.24, 29.14, 29.05, 28.93, 27.15, 26.52, 22.30, 14.15. IR (ATR): 3367, 3280, 2950, 2918, 2849, 1685, 1636, 1609, 1543, 1516, 1469, 1445, 1375, 1340, 1329, 1261, 1240, 1212, 1174, 1071, 952, 902, 835, 766, 722, 656, 609 cm^−1^. Elemental analysis for C_23_H_40_N_4_O_2_ (404.60); calculated: C, 68.28; H, 9.97; N, 13.85, found: C, 68.34; H, 10.03; N, 13.78. 

2-(4-Hydroxybenzoyl)-*N*-methylhydrazine-1-carboxamide **1aa**. White solid. Yield 30% (method A), mp 234–235 °C. ^1^H NMR (500 MHz, DMSO-*D*_6_) *δ* 10.04 (1H, s, OH), 9.85 (1H, s, NH), 7.78–7.72 (3H, m, NH, H2, H6), 6.82–7.78 (2H, m, H3, H5), 6.35 (1H, q, *J* = 4.7 Hz, NH-CH_3_), 2.56 (3H, d, *J* = 4.6 Hz, NH-CH_3_). ^13^C NMR (126 MHz, DMSO-*D*_6_) *δ* 166.32, 160.70, 159.33, 129.76, 123.56, 114.99, 26.44. IR (ATR): 3407, 3331, 3183, 1687, 1635, 1607, 1587, 1542, 1512, 1493, 1462, 1442, 1412, 1310, 1284, 1259, 1226, 1169, 1117, 1102, 976, 903, 844, 757, 688, 657, 634, 610 cm^−1^. Elemental analysis for C_9_H_11_N_3_O_3_ (209.21); calculated: C, 51.67; H, 5.30; N, 20.09, found: C, 51.64; H, 5.27; N, 20.01.

2-(4-Hydroxybenzoyl)-*N*-tridecylhydrazine-1-carboxamide **1bb**. White solid. Yield 25% (method B), 146–148 °C. ^1^H NMR (500 MHz, DMSO-*D*_6_) *δ* 10.43 (1H, s, OH), 9.83 (1H, s, NH), 7.99 (1H, s, NH), 7.82–7.79 (2H, m, H2, H6), 6.90–7.87 (2H, m, H3, H5), 6.42 (1H, t, *J* = 5.9 Hz, NH-CH_2_), 3.04 (2H, q, *J* = 6.4 Hz, NH-CH_2_), 1.37 (2H, p, *J* = 6.9 Hz, C^2^H_2_), 1.26–1.20 (20H, m, C^3^H_2_, C^4^H_2_, C^5^H_2_, C^6^H_2_, C^7^H_2_, C^8^H_2_, C^9^H_2_, C^10^H_2_, C^11^H_2_, C^12^H_2_), 0.84 (3H, t, *J* = 6.9 Hz, CH_3_). ^13^C NMR (126 MHz, DMSO-*D*_6_) *δ* 166.25, 160.78, 158.77, 129.70, 123.50, 115.01, 38.93, 31.53, 30.11, 29.31, 29.26, 29.08, 28.96, 28.78, 27.16, 26.55, 26.07, 22.33, 14.19. IR (ATR): 3392, 3197, 2955, 2919, 2850, 1660, 1639, 1613, 1582, 1501, 1468, 1378, 1327, 1291, 1270, 1246, 1172, 1074, 904, 847, 767, 721, 659, 652, 633 cm^−1^. Elemental analysis for C_21_H_35_N_3_O_3_ (377.53); calculated: C, 66.81; H, 9.34; N, 11.13, found: C, 66.75; H, 9.41; N, 11.02.

*N*-Methyl-2-(4-sulfamoylbenzoyl)hydrazine-1-carboxamide **1cc**. White solid. Yield 72% (method C), mp 233–234 °C. ^1^H NMR (600 MHz, DMSO-*D*_6_) *δ* 10.24 (1H, s, NH), 8.00 (2H, d, *J* = 8.2 Hz, H2, H6), 7.91 (1H, s, NH), 7.87 (2H, d, *J* = 8.3 Hz, H3, H5), 7.46 (2H, s, SO_2_NH_2_), 6.43 (2H, q, *J* = 4.6 Hz, NH-CH_3_), 2.54 (3H, d, *J* = 4.6 Hz, NH-CH_3_). ^13^C NMR (151 MHz, DMSO-*D*_6_) *δ* 165.99, 159.28, 147.16, 136.27, 128.82, 126.08, 26.79. IR (ATR): 904, 1015, 1074, 1098, 1120, 1162, 1187, 1291, 1314, 1416, 1473, 1541, 1567, 1647, 3100, 3241, 3360 cm^−1^. Elemental analysis for C_9_H_12_N_4_O_4_S (272.28); calculated: C, 39.70; H, 4.44; N, 20.58, found: C, 39.80; H, 4.44; N, 20.62. R*_f_*: 0.12.

2-(4-Sulfamoylbenzoyl)-*N*-tridecylhydrazine-1-carboxamide **1dd**. White solid. Yield 99% (method C), mp 116–118 °C. ^1^H NMR (600 MHz, DMSO-*D*_6_) *δ* 10.01 (1H, s, NH), 7.98 (2H, d, *J* = 8.2 Hz, H2, H6), 7.88 (2H, d, *J* = 8.2 Hz, H3, H5), 7.67 (1H, s, NH), 7.28 (2H, s, SO_2_NH_2_), 6.25 (1H, t, *J* = 5.9 Hz, NH-CH_2_), 3.01 (2H, q, *J* = 6.6 Hz, NH-CH_2_), 1.38 (2H, p, *J* = 6.9 Hz, C^2^H_2_), 1.25–1.21 (20H, m, C^3^H_2_, C^4^H_2_, C^5^H_2_, C^6^H_2_, C^7^H_2_, C^8^H_2_, C^9^H_2_, C^10^H_2_, C^11^H_2_, C^12^H_2_), 0.83 (3H, t, *J* = 6.9 Hz, CH_3_). ^13^C NMR (151 MHz, DMSO-*D*_6_) *δ* 165.86, 158.58, 147.30, 136.46, 128.62, 126.10, 31.75, 30.34, 29.50, 29.49, 29.48, 29.47, 29.46, 29.45, 29.29, 29.12, 26.84, 22.50, 14.28. IR (ATR): 676, 717, 748, 767, 853, 860, 901, 1022, 1093, 1163, 1257, 1289, 1326, 1343, 1377, 1472, 1478, 1519, 1572, 1647, 1667, 2850, 2918, 2954, 3288 cm^−1^. Elemental analysis for C_21_H_36_N_4_O_4_S (440.60); calculated: C, 57.25; H, 8.24; N, 12.72, found: C, 57.33; H, 8.20; N, 12.80. R*_f_*: 0.17.

2-Benzoyl-*N*-methylhydrazine-1-carboxamide **1ee**. White solid. Yield 28% (method A), mp 165–168 °C. ^1^H NMR (500 MHz, DMSO-*D*_6_) *δ* 10.10 (1H, s, NH), 7.91–7.86 (3H, m, NH, H2, H6), 7.57–7.53 (1H, m, H4), 7.49–7.45 (2H, m, H3, H5), 6.42 (2H, q, *J* = 4.7 Hz, NH-CH_3_), 2.58 (3H, d, *J* = 4.6 Hz, NH-CH_3_). ^13^C NMR (126 MHz, DMSO-*D*_6_) *δ* 166.58, 159.14, 132.96, 131.82, 128.46, 127.77, 26.45. IR (ATR): 3310, 3280, 3056, 1648, 1603, 1581, 1536, 1490, 1419, 1344, 1330, 1255, 1188, 1109, 1074, 1025, 982, 911, 804, 748, 684, 636, 616 cm^−1^. Elemental analysis for C_9_H_11_N_3_O_2_ (193.21); calculated: C, 55.95; H, 5.74; N, 21.75, found: C, 56.00; H, 5.84; N, 21.69.

2-Benzoyl-*N*-tridecylhydrazine-1-carboxamide **1ff**. White solid. Yield 34% (method B), mp 129–131 °C. ^1^H NMR (500 MHz, DMSO-*D*_6_) *δ* 10.11 (1H, s, NH), 7.83 (1H, s, NH), 7.65–7.61 (1H, m, H4), 7.56–7.51 (2H, m, H2, H6), 7.48–7.44 (2H, m, H3, H5), 6.53 (1H, t, *J* = 6.0 Hz, NH-CH_2_), 3.03 (2H, q, *J* = 6.5 Hz, NH-CH_2_), 1.36 (2H, p, *J* = 6.9 Hz, C^2^H_2_), 1.25–1.20 (20H, m, C^3^H_2_, C^4^H_2_, C^5^H_2_, C^6^H_2_, C^7^H_2_, C^8^H_2_, C^9^H_2_, C^10^H_2_, C^11^H_2_, C^12^H_2_), 0.84 (3H, t, *J* = 6.9 Hz, CH_3_). ^13^C NMR (126 MHz, DMSO-*D*_6_) *δ* 165.96, 158.55, 132.96, 131.78, 128.88, 127.96, 45.43, 31.50, 30.08, 29.27, 29.23, 29.14, 29.05, 28.92, 26.53, 26.04, 22.30, 14.16, 8.58. IR (ATR): 3300, 2948, 2918, 2849, 1668, 1645, 1604, 1582, 1540, 1500, 1469, 1343, 1330, 1260, 1189, 1124, 1069, 1017, 982, 912, 806, 747, 684, 638, 618 cm^−1^. Elemental analysis for C_21_H_35_N_3_O_2_ (361.53); calculated: C, 69.77; H, 9.76; N, 11.62, found: C, 69.84; H, 9.83; N, 11.69.

*N*-Methyl-2-[3-(trifluoromethyl)benzoyl]hydrazine-1-carboxamide **2a**. White solid. Yield 21% (method A), mp 202–204 °C. ^1^H NMR (500 MHz, DMSO-*D*_6_) *δ* 10.38 (1H, s, NH), 8.23 (1H, t, *J* = 1.9 Hz, H2), 8.19–8.16 (1H, m, H6), 7.96 (1H, s, NH), 7.93–7.90 (1H, m, H4), 7.73 (1H, t, *J* = 7.8 Hz, H5), 6.51 (1H, q, *J* = 4.5 Hz, NH-CH_3_), 2.58 (3H, d, *J* = 4.6 Hz, NH-CH_3_). ^13^C NMR (126 MHz, DMSO-*D*_6_) δ 164.99, 158.92, 134.16, 131.82, 129.76, 129.26 (q, *J* = 32.0 Hz), 128.19 (q, *J* = 3.8 Hz), 124.41 (q, *J* = 3.9 Hz), 124.18 (q, *J* = 272.3 Hz), 26.40. IR (ATR): 3235, 3049, 1655, 1617, 1581, 1548, 1488, 1421, 1348, 1326, 1312, 1288, 1259, 1220, 1163, 1116, 1075, 983, 923, 819, 800, 762, 698, 667, 649, 615 cm^−1^. Elemental analysis for C_10_H_10_F_3_N_3_O_2_ (261.20); calculated: C, 45.98; H, 3.86; N, 16.09, found: C, 46.07; H, 3.90; N, 16.08.

*N*-Tridecyl-2-[3-(trifluoromethyl)benzoyl]hydrazine-1-carboxamide **2b**. White solid. Yield 95% (method C), mp 140–141 °C. ^1^H NMR (600 MHz, DMSO-*D*_6_) *δ* 10.16 (1H, s, NH), 8.18 (1H, s, NH), 8.16–8.12 (1H, m, H2), 7.88–7.84 (1H, m, H6), 7.71–7.66 (2H, m, H4, H5), 6.29 (1H, t, *J* = 6.1 Hz, NH-CH_2_), 3.01 (2H, q, *J* = 6.6 Hz, NH-CH_2_), 1.38 (2H, p, *J* = 6.9 Hz, C^2^H_2_), 1.25–1.21 (20H, m, C^3^H_2_, C^4^H_2_, C^5^H_2_, C^6^H_2_, C^7^H_2_, C^8^H_2_, C^9^H_2_, C^10^H_2_, C^11^H_2_, C^12^H_2_), 0.83 (3H, t, *J* = 6.7 Hz, CH_3_). ^13^C NMR (151 MHz, DMSO-*D*_6_) δ 165.49, 158.62, 134.55, 132.05, 130.04, 129.73 (q, *J* = 31.8 Hz), 128.15 (q, *J* = 3.9 Hz), 124.55 (q, *J* = 272.3 Hz), 124.73 (q, *J* = 4.7 Hz), 31.75, 30.33, 29.49, 29.48, 29.47, 29.46, 29.45, 29.44, 29.29, 29.11, 26.83, 22.49, 14.25. IR (ATR): 663, 725, 817, 922, 1073, 1124, 1170, 1244, 1258, 1287, 1311, 1326, 1468, 1558, 1568, 1650, 1668, 1688, 2851, 2921, 3278 cm^−1^. Elemental analysis for C_22_H_34_F_3_N_3_O_2_ (429.53); calculated: C, 61.52; H, 7.98; N, 9.78, found: C, 61.67; H, 8.09; N, 9.88. R*_f_*: 0.48.

*N*-Methyl-2-[2-(trifluoromethyl)benzoyl]hydrazine-1-carboxamide **2c**. White solid. Yield 21% (method A), mp 196–198 °C. ^1^H NMR (500 MHz, DMSO-*D*_6_) *δ* 10.38 (1H, s, NH), 8.23 (1H, s, NH), 8.17 (1H, d, *J* = 7.9 Hz, H6), 7.96–7.91 (2H, m, H3, H4), 7.74 (1H, t, *J* = 7.8 Hz, H5), 6.51 (1H, q, *J* = 4.6 Hz, NH-CH_3_), 2.58 (3H, d, *J* = 4.5 Hz, NH-CH_3_). ^13^C NMR (126 MHz, DMSO-*D*_6_) δ 165.24, 158.96, 133.92, 131.87, 129.89, 129.29 (q, *J* = 32.2 Hz), 128.35 (q, *J* = 3.8 Hz), 124.46 (q, *J* = 4.0 Hz), 124.15 (q, *J* = 272.2 Hz), 26.40. IR (ATR): 3369, 3253, 3050, 1654, 1617, 1582, 1548, 1421, 1348, 1326, 1313, 1259, 1220, 1163, 1116, 1075, 986, 923, 819, 801, 762, 698, 667, 649, 611 cm^−1^. Elemental analysis for C_10_H_10_F_3_N_3_O_2_ (261.20); calculated: C, 45.98; H, 3.86; N, 16.09, found: C, 46.02; H, 3.93; N, 16.12.

*N*-Tridecyl-2-[2-(trifluoromethyl)benzoyl]hydrazine-1-carboxamide **2d**. White solid. Yield 92% (method C), mp 134–136 °C. ^1^H NMR (600 MHz, DMSO-*D*_6_) *δ* 9.82 (1H, s, NH), 7.78–7.73 (2H, m, H3, H6), 7.72–7.68 (1H, m, H4), 7.66–7.61 (2H, m, NH, H5), 5.98 (1H, t, *J* = 5.8 Hz, NH-CH_2_), 3.03 (2H, q, *J* = 6.6 Hz, NH-CH_2_), 1.40 (2H, p, *J* = 7.0 Hz, C^2^H_2_), 1.26–1.23 (20H, m, C^3^H_2_, C^4^H_2_, C^5^H_2_, C^6^H_2_, C^7^H_2_, C^8^H_2_, C^9^H_2_, C^10^H_2_, C^11^H_2_, C^12^H_2_), 0.83 (3H, t, *J* = 6.8 Hz, CH_3_). ^13^C NMR (151 MHz, DMSO-*D*_6_) *δ* 167.28, 158.31, 134.99, 132.72, 130.68, 129.74, 127.15 (q, *J* = 31.8 Hz), 126.77 (q, *J* = 4.2 Hz), 124.19 (q, *J* = 273.7 Hz), 31.75, 30.31, 29.50, 29.49, 29.48, 29.47, 29.46, 29.45, 29.28, 29.12, 26.82, 22.49, 14.26. IR (ATR): 665, 727, 765, 900, 1036, 1056, 1110, 1120, 1140, 1188, 1240, 1256, 1271, 1315, 1378, 1448, 1463, 1538, 1588, 1602, 1613, 1660, 2849, 2924, 2954, 3180 cm^−1^. Elemental analysis for C_22_H_34_F_3_N_3_O_2_ (429.53); calculated: C, 61.52; H, 7.98; N, 9.78, found: C, 61.49; H, 8.10; N, 9.84. R*_f_*: 0.38.

*N*-Methyl-2-(3-nitrobenzoyl)hydrazine-1-carboxamide **2e**. Pale yellow solid. Yield 33% (method A), mp 220–222 °C. ^1^H NMR (600 MHz, DMSO-*D*_6_) *δ* 10.44 (1H, s, NH), 8.68 (1H, t, *J* = 2.0 Hz, H2), 8.38 (1H, dd, *J* = 8.2, 2.4 Hz, H4), 8.29–8.26 (1H, m, H6), 7.96 (1H, s, NH), 7.77 (1H, t, *J* = 8.0 Hz, H5), 6.50 (1H, s, NH-CH_3_), 2.55 (3H, d, *J* = 4.5 Hz, NH-CH_3_). ^13^C NMR (151 MHz, DMSO-*D*_6_) *δ* 165.02, 159.23, 148.23, 134.87, 134.52, 130.68, 126.75, 122.95, 26.76. IR (ATR): 3305, 3099, 2949, 1668, 1644, 1587, 1542, 1519, 1476, 1437, 1416, 1354, 1340, 1257, 1228, 1161, 1130, 1086, 985, 935, 827, 777, 726, 673, 652, 644, 606 cm^−1^. Elemental analysis for C_9_H_10_N_4_O_4_ (238.20); calculated: C, 45.38; H, 4.23; N, 23.52, found: C, 45.41; H, 4.30; N, 23.46. 

2-(3-Nitrobenzoyl)-*N*-tridecylhydrazine-1-carboxamide **2f**. Pale yellow solid. Yield 64% (method C), mp 146–147 °C. ^1^H NMR (600 MHz, DMSO-*D*_6_) *δ* 10.40 (1H, s, NH), 8.67 (1H, t, *J* = 2.0 Hz, H2), 8.36 (1H, dd, *J* = 8.1, 2.3 Hz, H4), 8.27 (1H, dd, *J* = 8.00, 1.4 Hz, H6), 7.82 (1H, s, NH), 7.76 (1H, t, *J* = 8.0 Hz, H5), 6.46 (1H, t, *J* = 5.9 Hz, NH-CH_2_), 2.99 (2H, q, *J* = 6.6 Hz, NH-CH_2_), 1.36 (2H, p, *J* = 7.0 Hz, C^2^H_2_), 1.24–1.18 (20H, m, C^3^H_2_, C^4^H_2_, C^5^H_2_, C^6^H_2_, C^7^H_2_, C^8^H_2_, C^9^H_2_, C^10^H_2_, C^11^H_2_, C^12^H_2_), 0.82 (3H, t, *J* = 6.9 Hz, CH_3_). ^13^C NMR (151 MHz, DMSO-*D*_6_) *δ* 164.84, 158.60, 148.32, 135.01, 134.42, 130.64, 126.63, 122.88, 31.80, 30.38, 29.57, 29.56, 29.55, 29.54, 29.52, 29.50, 29.34, 29.20, 26.84, 22.58, 14.41. IR (ATR): 629, 720, 820, 924, 1084, 1238, 1256, 1346, 1467, 1482, 1526, 1539, 1583, 1566, 1613, 2850, 2925, 2955, 3223, 3351 cm^−1^. Elemental analysis for C_21_H_34_N_4_O_4_ (406.53); calculated: C, 62.05; H, 8.43; N, 13.78, found: C, 62.14; H, 8.31; N, 13.84. R*_f_*: 0.48.

*N*-Methyl-2-(2-nitrobenzoyl)hydrazine-1-carboxamide **2g**. Pale yellow solid. Yield 17% (method A), mp 200–202 °C. ^1^H NMR (600 MHz, DMSO-*D*_6_) *δ* 10.50 (1H, s, NH), 8.09 (1H, d, *J* = 8.1 Hz, H3), 8.03 (1H, s, NH), 7.80 (1H, t, *J* = 7.5 Hz, H5), 7.71–7.64 (2H, m, H4, H6), 6.15 (1H, q, *J* = 4.6 Hz, NH-CH_3_), 2.53 (3H, d, *J* = 4.5 Hz, NH-CH_3_). ^13^C NMR (151 MHz, DMSO-*D*_6_) *δ* 164.92, 159.03, 143.84, 135.50, 133.28, 131.12, 129.80, 124.97, 26.78. IR (ATR): 3355, 3103, 2954, 1671, 1644, 1585, 1540, 1524, 1477, 1356, 1255, 1232, 1061, 901, 935, 827, 770, 729, 671, 649 cm^−1^. Elemental analysis for C_9_H_10_N_4_O_4_ (238.20); calculated: C, 45.38; H, 4.23; N, 23.52, found: C, 45.42; H, 4.17; N, 23.56. 

2-(2-Nitrobenzoyl)-*N*-tridecylhydrazine-1-carboxamide **2h**. Pale yellow solid. Yield 21% (method B), mp 148–150 °C. ^1^H NMR (600 MHz, DMSO-*D*_6_) *δ* 10.21 (1H, s, NH), 8.03 (1H, d, *J* = 8.1 Hz, H3), 8.00 (1H, s, NH), 7.80 (1H, t, *J* = 7.5 Hz, H5), 7.73–7.67 (2H, m, H4, H6), 6.15 (1H, t, *J* = 5.8 Hz, NH-CH_2_), 3.00 (2H, q, *J* = 6.6 Hz, NH-CH_2_), 1.36 (2H, p, *J* = 6.8 Hz, C^2^H_2_), 1.24–1.18 (20H, m, C^3^H_2_, C^4^H_2_, C^5^H_2_, C^6^H_2_, C^7^H_2_, C^8^H_2_, C^9^H_2_, C^10^H_2_, C^11^H_2_, C^12^H_2_), 0.81 (3H, t, *J* = 6.9 Hz, CH_3_). ^13^C NMR (151 MHz, DMSO-*D*_6_) δ 165.84, 158.28, 147.70, 134.18, 131.86, 131.00, 130.19, 124.75, 39.61, 31.83, 30.33, 29.60, 26.59, 29.56, 29.55, 29.36, 29.30, 29.25, 26.82, 22.63, 14.48. IR (ATR): 3356, 3179, 2955, 2924, 2489, 1608, 1585, 1539, 1524, 1478, 1460, 1352, 1255, 1238, 1064, 901, 859, 786, 757, 728, 698, 666 cm^−1^. Elemental analysis for C_21_H_34_N_4_O_4_ (406.53); calculated: C, 62.05; H, 8.43; N, 13.78, found: C, 62.10; H, 8.41; N, 13.75. 

2-(3-Bromobenzoyl)-*N*-methylhydrazine-1-carboxamide **2i**. White solid. Yield 65% (method A), mp 190–192 °C. ^1^H NMR (600 MHz, DMSO-*D*_6_) δ 10.18 (1H, s, NH), 8.04 (1H, t, *J* = 1.9 Hz, H2), 7.88–7.81 (2H, m, NH, H6), 7.74–7.71 (1H, m, H4), 7.42 (1H, t, *J* = 7.9 Hz, H5), 6.44 (1H, s, NH-CH_3_), 2.54 (3H, d, *J* = 4.5 Hz, NH-CH_3_). ^13^C NMR (151 MHz, DMSO-*D*_6_) δ 165.58, 159.31, 135.55, 134.87, 131.10, 130.86, 127.23, 122.12, 26.76. IR (ATR): 3289, 1670, 1646, 1588, 1532, 1489, 1470, 1416, 1337, 1261, 1224, 1072, 995, 920, 808, 752, 724, 678, 644, 631, 616 cm^−1^. Elemental analysis for C_9_H_10_BrN_3_O_2_ (277.10); calculated: C, 39.73; H, 3.70; N, 15.44, found: C, 39.74; H, 3.76; N, 15.51.

2-(3-Bromobenzoyl)-*N*-tridecylhydrazine-1-carboxamide **2j**. White solid. Yield 43% (method B), mp 145–147 °C. ^1^H NMR (600 MHz, DMSO-*D*_6_) δ 10.16 (1H, s, NH), 8.03 (1H, s, H2), 7.83 (1H, d, *J* = 7.8 Hz, H6), 7.78 (1H, s, NH), 7.72 (1H, d, *J* = 8.0 Hz, H4), 7.41 (1H, t, *J* = 7.9 Hz, H5), 6.47 (1H, t, *J* = 5.9 Hz, NH-CH_2_), 2.96 (2H, q, *J* = 6.6 Hz, NH-CH_2_), 1.34 (2H, p, *J* = 6.8 Hz, C^2^H_2_), 1.26–1.21 (20H, m, C^3^H_2_, C^4^H_2_, C^5^H_2_, C^6^H_2_, C^7^H_2_, C^8^H_2_, C^9^H_2_, C^10^H_2_, C^11^H_2_, C^12^H_2_), 0.81 (3H, t, *J* = 6.9 Hz, CH_3_). ^13^C NMR (151 MHz, DMSO-*D*_6_) δ 165.45, 158.74, 135.54, 134.86, 131.10, 130.83, 127.18, 122.13, 39.73, 31.84, 30.40, 29.63, 29.61, 29.56, 29.54, 29.48, 29.38, 29.26, 26.84, 22.64, 14.48. IR (ATR): 3322, 3038, 2921, 2850, 1660, 1650, 1588, 1566, 1468, 1336, 1257, 1244, 1070, 997, 924, 901, 805, 724, 683, 659, 644, 632, 613 cm^−1^. Elemental analysis for C_21_H_34_BrN_3_O_2_ (440.43); calculated: C, 57.27; H, 7.78; N, 9.54, found: C, 57.24; H, 7.74; N, 9.59.

2-(2-Bromobenzoyl)-*N*-methylhydrazine-1-carboxamide **2k**. White solid. Yield 20% (method A), mp 180–182 °C. ^1^H NMR (600 MHz, DMSO-*D*_6_) δ 9.93 (1H, d, *J* = 2.1 Hz, NH), 8.01 (1H, d, *J* = 2.1 Hz, NH), 7.63 (1H, dd, *J* = 8.0, 1.8 Hz, H6), 7.52 (1H, dd, *J* = 7.8, 1.8 Hz, H3), 7.42 (1H, td, *J* = 7.8, 1.8 Hz, H4), 7.36 (1H, td, *J* = 7.8, 1.7 Hz, H5), 6.14 (1H, q, *J* = 4.6 Hz, NH-CH_3_), 2.58 (3H, d, *J* = 4.6 Hz, NH-CH_3_). ^13^C NMR (151 MHz, DMSO-*D*_6_) δ 167.64, 158.96, 137.33, 133.41, 132.00, 130.13, 127.99, 119.93, 26.84. IR (ATR): 3385, 3284, 3188, 3002, 1710, 1645, 1591, 1568, 1542, 1472, 1429, 1412, 1356, 1327, 1251, 1147, 1123, 1029, 972, 901, 772, 747, 724, 701, 663, 646, 628 cm^−1^. Elemental analysis for C_9_H_10_BrN_3_O_2_ (277.10); calculated: C, 39.73; H, 3.70; N, 15.44, found: C, 39.70; H, 3.72; N, 15.39.

2-(2-Bromobenzoyl)-*N*-tridecylhydrazine-1-carboxamide **2l**. White solid. Yield 67% (method B), mp 120–121 °C. ^1^H NMR (500 MHz, DMSO-*D*_6_) δ 9.96 (1H, d, *J* = 2.1 Hz, NH), 7.96 (1H, d, *J* = 2.1 Hz, NH), 7.67 (1H, d, *J* = 8.0, H6), 7.51 (1H, dd, *J* = 7.6, 1.8 Hz, H3), 7.46 (1H, td, *J* = 7.8, 1.8 Hz, H4), 7.39 (1H, td, *J* = 7.8, 1.9 Hz, H5), 6.20 (1H, t, *J* = 5.8 Hz, NH-CH_2_), 3.03 (2H, q, *J* = 6.6 Hz, NH-CH_2_), 1.42–1.36 (2H, m, C^2^H_2_), 1.28–1.20 (20H, m, C^3^H_2_, C^4^H_2_, C^5^H_2_, C^6^H_2_, C^7^H_2_, C^8^H_2_, C^9^H_2_, C^10^H_2_, C^11^H_2_, C^12^H_2_), 0.85 (3H, t, *J* = 6.7 Hz, CH_3_). ^13^C NMR (126 MHz, DMSO-*D*_6_) δ 167.17, 157.97, 136.97, 133.01, 131.61, 129.67, 127.64, 119.51, 45.78, 31.46, 30.01, 29.26, 29.24, 29.21, 29.19, 29.00, 28.88, 26.48, 26.33, 22.26, 14.12. IR (ATR): 3354, 3201, 2954, 2924, 2849, 1607, 1591, 1573, 1591, 1541, 1478, 1467, 1456, 1257, 1237, 1223, 1027, 984, 899, 777, 756, 741, 724, 654, 636 cm^−1^. Elemental analysis for C_21_H_34_BrN_3_O_2_ (440.43); calculated: C, 57.27; H, 7.78; N, 9.54, found: C, 57.20; H, 7.80; N, 9.49.

*N*-Methyl-2-{2-[4-(trifluoromethyl)phenyl]acetyl}hydrazine-1-carboxamide **3a**. White solid. Yield 50% (method A), mp 209–210 °C. ^1^H NMR (600 MHz, DMSO-*D*_6_) *δ* 9.73 (1H, d, *J* = 2.0 Hz, NH), 7.76 (1H, d, *J* = 2.0 Hz, NH), 7.63 (2H, d, *J* = 8.1 Hz, H3, H5), 7.47 (2H, d, *J* = 7.9 Hz, H2, H6), 6.27 (1H, q, *J* = 4.9 Hz, NH-CH_3_), 3.52 (2H, s, CH_2_), 2.52 (3H, d, *J* = 4.5 Hz, NH-CH_3_). ^13^C NMR (151 MHz, DMSO-*D*_6_) *δ* 169.92, 159.12, 141.18, 130.56, 127.78 (q, *J* = 31.9 Hz), 125.54 (q, *J* = 3.9 Hz), 124.93 (q, *J* = 273.6 Hz), 42.49, 26.72. IR (ATR): 3310, 3269, 3072, 3033, 1655, 1644, 1583, 1544, 1509, 1425, 1325, 1314, 1260, 1168, 1116, 1070, 1015, 986, 914, 858, 777, 765, 743, 693, 644, 629 cm^−1^. Elemental analysis for C_11_H_12_F_3_N_3_O_2_ (275.23); calculated: C, 48.00; H, 4.39; N, 15.27, found: C, 47.99; H, 4.31; N, 15.33. 

*N*-Tridecyl-2-{2-[4-(trifluoromethyl)phenyl]acetyl}hydrazine-1-carboxamide **3b**. White solid. Yield 25% (method B), mp 154–156 °C. ^1^H NMR (500 MHz, DMSO-*D*_6_) *δ* 9.77 (1H, s, NH), 7.71 (1H, s, NH), 7.65 (2H, d, *J* = 7.9 Hz, H3, H5), 7.51 (2H, d, *J* = 7.9 Hz, H2, H6), 6.29 (1H, t, *J* = 5.8 Hz, NH-CH_2_), 3.55 (2H, s, CH_2_), 2.97 (2H, q, *J* = 6.6 Hz, NH-CH_2_), 1.34 (2H, p, *J* = 7.4 Hz, C^2^H_2_), 1.26–1.20 (20H, m, C^3^H_2_, C^4^H_2_, C^5^H_2_, C^6^H_2_, C^7^H_2_, C^8^H_2_, C^9^H_2_, C^10^H_2_, C^11^H_2_, C^12^H_2_), 0.84 (3H, t, *J* = 6.8 Hz, CH_3_). ^13^C NMR (126 MHz, DMSO-*D*_6_) *δ* 169.42, 158.13, 140.86, 130.15, 127.42 (q, *J* = 31.8 Hz), 125.14 (q, *J* = 4.0 Hz), 124.56 (q, *J* = 271.9 Hz), 40.03, 37.48, 31.47, 30.00, 29.24, 29.22, 29.20, 29.01, 28.89, 26.47, 22.27, 14.10. IR (ATR): 3311, 2950, 2918, 2849, 1667, 1639, 1569, 1558, 1508, 1489, 1469, 1417, 1326, 1243, 1165, 1119, 1068, 1020, 981, 873, 827, 723, 652, 644, 635, 626 cm^−1^. Elemental analysis for C_23_H_36_F_3_N_3_O_2_ (443.56); calculated: C, 62.28; H, 8.18; N, 9.47, found: C, 62.29; H, 8.10; N, 9.55.

*N*-Methyl-2-{[4-(trifluoromethyl)phenyl]sulfonyl}hydrazine-1-carboxamide **3c**. White solid. Yield 64% (method C), mp 209–210 °C (decomp.). ^1^H NMR (600 MHz, DMSO-*D*_6_) *δ* 9.69 (1H, s, NH), 8.04 (1H, s, NH), 7.99–7.91 (4H, m, H2, H3, H5, H6), 6.36 (1H, q, *J* = 4.6 Hz, NH-CH_3_), 2.47 (3H, d, *J* = 4.6 Hz, NH-CH_3_). ^13^C NMR (151 MHz, DMSO-*D*_6_) *δ* 158.61, 142.87, 133.15 (q, *J* = 32.1 Hz), 129.32, 126.71 (q, *J* = 4.4 Hz), 124.11 (q, *J* = 272.9 Hz), 26.74. IR (ATR): 626, 707, 762, 793, 845, 964, 1016, 1062, 1095, 1110, 1135, 1172, 1192, 1302, 1319, 1358, 1405, 1418, 1489, 1564, 1648, 3113, 3417 cm^−1^. Elemental analysis for C_9_H_10_F_3_N_3_O_3_S (297.25); calculated: C, 36.37; H, 3.39; N, 14.14, found: C, 36.49; H, 3.30; N, 14.13. R*_f_*: 0.43.

*N*-Tridecyl-2-{[4-(trifluoromethyl)phenyl]sulfonyl}hydrazine-1-carboxamide **3d**. White solid. Yield 96% (method C), mp 157–158 °C. ^1^H NMR (600 MHz, DMSO-*D*_6_) *δ* 9.67 (1H, s, NH), 8.02 (1H, s, NH), 7.97 (2H, d, *J* = 8.3 Hz, H3, H5), 7.92 (2H, d, *J* = 8.3 Hz, H2, H6), 6.28 (1H, t, *J* = 6.0 Hz, NH-CH_2_), 2.85 (2H, q, *J* = 6.8 Hz, NH-CH_2_), 1.22–1.13 (22H, m, C^2^H_2_, C^3^H_2_, C^4^H_2_, C^5^H_2_, C^6^H_2_, C^7^H_2_, C^8^H_2_, C^9^H_2_, C^10^H_2_, C^11^H_2_, C^12^H_2_), 0.81 (3H, t, *J* = 6.9 Hz, CH_3_). ^13^C NMR (151 MHz, DMSO-*D*_6_) *δ* 157.75, 142.92, 133.13 (q, *J* = 32.1 Hz), 129.36, 126.61 (q, *J* = 4.5 Hz), 124.11 (q, *J* = 272.9 Hz), 31.83, 30.16, 29.60, 29.59, 29.58, 29.57, 29.56, 29.55, 29.29, 29.24, 26.70, 22.62, 14.45. IR (ATR): 635, 717, 744, 790, 842, 892, 1018, 1065, 1094, 1111, 1142, 1161, 1172, 1316, 1330, 1406, 1471, 1489, 1549, 1636, 1655, 2850, 2917, 2955, 3342 cm^−1^. Elemental analysis for C_21_H_34_F_3_N_3_O_3_S (465.58); calculated: C, 54.18; H, 7.36; N, 9.03, found: C, 54.29; H, 7.47; N, 8.92. R*_f_*: 0.56.

### 3.2. Inhibition of Acetylcholinesterase and Butyrylcholinesterase

The inhibitory activity of **1–3** for AChE and BChE was determined spectrophotometrically using modified Ellman’s method [25] to quantify them in terms of IC_50_ values.

The enzyme activity in the final reaction mixture (2000 µL) was 0.2 U/mL, the concentration of ATCh (or BTCh) was 40 µM, and the concentration of DTNB was 100 µM for all reactions. The investigated derivatives were dissolved in DMSO and then diluted with demineralized water (conductivity 3 μS, equipment supplier BKG Water Treatment, Hradec Králové, Czech Republic) to a concentration of 1000 µM. Five different inhibitor concentrations were used for all tested compounds in the final reaction mixture. The final concentration of DMSO was 0.2%. All experiments were performed in triplicate. The average values of the reaction rate (v_0_-uninhibited reaction, v_i_-inhibited reaction) were used to construct the dependence of v_0_/v_i_ on the concentration of inhibitors. IC_50_ values were calculated from obtained regression curve equations.

Acetylcholinesterase originating from electric eels (*Electrophorus electricus* L.; *Ee*AChE) and butyrylcholinesterase obtained from equine serum (EqBChE) were used. The clinically used drug rivastigmine was involved as a reference dual AChE and BChE inhibitor. All enzymes, substrates and rivastigmine were purchased from Merck (Prague, Czech Republic).

### 3.3. Molecular Docking

The crystallographic structures of human AChE and human BChE were obtained from a protein data bank (www.rcsb.org, accessed on 14 December 2022; pdb codes 4PQE and 1P0I, respectively). The 3D structures of ligands were prepared in Chem3D Pro 19.1 (ChemBioOffice 2019 Package, CambridgeSoft, Cambridge, MA, USA). In the preparation process, all water molecules were removed from the enzymes, and structures of enzymes and ligands were optimized using the UCSF Chimera software package (Amber force field) [26]. Docking was performed using Autodock Vina [27] and Autodock 4.2 [28] (a Lamarckian genetic algorithm was used). The 3D affinity grid box was designed to include the full active and peripheral site of AChE and BChE. The number of grid points in the x-, y- and z-axes was 20, 20 and 20, with grid points separated by 1 Å (Autodock Vina), and 40, 40 and 40, with grid points separated by 0.4 Å (Autodock 4). The flexibility of several protein side chains was allowed. Graphic visualizations of ligand–enzyme interactions were prepared in PyMOL (The PyMOL Molecular Graphics System, Version 1.5 Schrödinger, LLC).

## 4. Conclusions

A series of forty-eight *N*-methyl/tridecyl-2-aryloylhydrazine-1-carboxamides and their analogs were designed, synthesized, characterized and investigated as potential inhibitors of acetylcholinesterase and butyrylcholinesterase. Almost all of them exhibited the dual inhibition of both cholinesterases, with IC_50_ values in the micromolar range. Their activity against AChE was generally higher than that against BChE, with a narrower range of IC_50_ values. Some of them achieved better or fairly comparable IC_50_ values to the drug rivastigmine. Molecular docking showed details of the molecular interactions of inhibitors with enzymes that differed for AChE and BChE. 2-(4-Nitrobenzoyl)-*N*-tridecylhydrazine-1-carboxamide was identified as the most promising molecule in terms of the inhibition of both enzymes at low concentrations and lipophilicity.

Further biological activity (especially antimicrobial and anticancer) will be investigated in the near future.

## Figures and Tables

**Table 1:** Comparison of yields of different synthetic methods.

Method	Yields [%]
A (MIC substitute)	17–65
B (amine + triphosgene)	21–84
C (Curtius rearrangement)	64–99

**Table 2:** Inhibition of AChE and BChE, selectivity indexes and lipophilicity of hydrazinecarboxamides **1–3**.

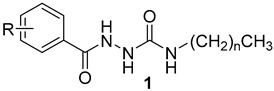						
Code	R	*n*	IC_50_ AChE [µM]	IC_50_ BChE [µM]	Selectivity BChE/AChE	log*P* _o/w_
**P1**	4-CF_3_	0	**31.23 ± 1.87**	84.16 ± 2.10	2.7	1.69
**P2**	4-CF_3_	12	**28.90 ± 0.67**	277.48 ± 10.27	9.6	5.87
Series 1: replacing 4-CF_3_ group						
**1a**	4-OCF_3_	0	56.76 ± 0.49	204.25 ± 13.51	3.6	1.49
**1b**	4-OCF_3_	12	65.14 ± 1.15	125.23 ± 1.17	1.9	5.84
**1c**	4-F	0	57.23 ± 1.66	366.27 ± 78.43	6.4	0.82
**1d**	4-F	12	60.16 ± 0.53	483.50 ± 20.19	8.0	5.25
**1e**	4-Cl	0	**44.08 ± 1.79**	319.49 ± 12.64	7.2	1.18
**1f**	4-Cl	12	86.59 ± 0.29	88.68 ± 3.28	1.0	5.54
**1g**	4-Br	0	50.60 ± 1.55	359.58 ± 2.17	7.1	1.27
**1h**	4-Br	12	72.66 ± 0.67	**27.08 ± 0.07**	**0.4**	5.56
**1i**	4-I	0	73.76 ± 1.78	>500	>6.8	1.28
**1j**	4-I	12	79.73 ± 2.08	192.08 ± 3.45	2.4	5.59
**1k**	4-NO_2_	0	53.99 ± 2.21	177.78 ± 9.13	3.3	0.07
**1l**	4-NO_2_	12	53.69 ± 1.20	**22.31 ± 0.09**	**0.4**	4.29
**1m**	4-CN	0	55.64 ± 0.27	447.92 ± 39.03	8.1	0.36
**1n**	4-CN	12	54.59 ± 1.27	417.57 ± 15.72	7.6	4.66
**1o**	4-Me	0	62.29 ± 0.56	284.42 ± 13.16	4.6	0.98
**1p**	4-Me	12	100.08 ± 2.10	277.38 ± 6.50	2.8	5.16
**1q**	4-*t*-Bu	0	59.97 ± 2.47	281.35 ± 7.79	4.7	1.85
**1r**	4-*t*-Bu	12	75.25 ± 0.43	57.24 ± 0.23	0.8	6.12
**1s**	4-Ph	0	64.42 ± 1.67	301.34 ± 7.46	4.7	2.03
**1t**	4-Ph	12	51.14 ± 0.98	**32.10 ± 0.18**	0.6	6.26
**1u**	4-OMe	0	57.16 ± 0.92	170.62 ± 8.36	3.0	0.63
**1v**	4-OMe	12	83.63 ± 3.16	376.22 ± 17.00	4.5	4.88
**1w**	4-OPh	0	74.99 ± 0.84	302.31 ± 2.11	4.0	1.72
**1x**	4-OPh	12	58.29 ± 3.29	367.76 ± 14.28	6.3	6.11
**1y**	4-NMe_2_	0	70.03 ± 1.74	299.89 ± 13.64	4.3	0.68
**1z**	4-NMe_2_	12	87.67 ± 0.83	188.33 ± 2.22	2.1	4.87
**1aa**	4-OH	0	67.57 ± 1.30	247.03 ± 18.39	3.7	0.16
**1bb**	4-OH	12	74.72 ± 3.31	148.61 ± 10.73	2.0	4.41
**1cc**	4-SO_2_NH_2_	0	86.14 ± 0.50	250.42 ± 5.22	2.9	-0.52
**1dd**	4-SO_2_NH_2_	12	74.58 ± 1.12	274.63 ± 10.70	3.7	3.84
**1ee**	H	0	63.25 ± 1.49	229.21 ± 0.08	3.6	0.48
**1ff**	H	12	73.40 ± 2.45	234.84 ± 5.57	3.2	4.83
Series 2: positional isomers						
**2a**	3-CF_3_	0	61.33 ± 1.00	225.13 ± 7.84	3.7	1.83
**2b**	3-CF_3_	12	63.92 ± 0.83	292.39 ± 10.67	4.6	5.97
**2c**	2-CF_3_	0	61.09 ± 0.03	296.23 ± 0.87	4.8	1.45
**2d**	2-CF_3_	12	80.44 ± 2.10	422.06 ± 12.40	5.2	5.97
**2e**	3-NO_2_	0	67.39 ± 1.72	>500	>7.4	0.02
**2f**	3-NO_2_	12	62.46 ± 1.79	236.33 ± 6.54	3.8	4.31
**2g**	2-NO_2_	0	64.32 ± 2.23	191.41 ± 10.08	3.0	−0.24
**2h**	2-NO_2_	12	54.88 ± 1.08	>500	**>9.1**	4.29
**2i**	3-Br	0	51.86 ± 2.00	353.80 ± 8.79	6.8	1.25
**2j**	3-Br	12	**44.34 ± 0.10**	>500	**>11.3**	5.49
**2k**	2-Br	0	59.40 ± 1.83	>500	>8.4	1.05
**2l**	2-Br	12	53.99 ± 0.44	>500	**>9.3**	5.50
Series 3: modification of the linker						
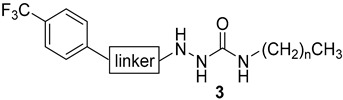						
**3a**	CH_2_CO	0	68.51 ± 0.25	131.04 ± 9.70	1.9	1.74
**3b**	CH_2_CO	12	81.69 ± 0.45	292.90 ± 1.72	3.6	5.98
**3c**	SO_2_	0	71.14 ± 0.94	424.74 ± 13.22	7.0	1.26
**3d**	SO_2_	12	58.78 ± 0.96	338.98 ± 11.56	8.5	5.46
	Rivastigmine		56.10 ± 1.41	38.40 ± 1.97	-	0.7

IC_50_ values are expressed as the mean ± SD from three independent experiments. The lowest IC_50_ values for each enzyme are given in bold as well as the most selective inhibitors for both enzymes.

## Data Availability

Data is contained within the article.
